# Quantitative spatial analysis of haematopoiesis-regulating stromal cells in the bone marrow microenvironment by 3D microscopy

**DOI:** 10.1038/s41467-018-04770-z

**Published:** 2018-06-28

**Authors:** Alvaro Gomariz, Patrick M. Helbling, Stephan Isringhausen, Ute Suessbier, Anton Becker, Andreas Boss, Takashi Nagasawa, Grégory Paul, Orcun Goksel, Gábor Székely, Szymon Stoma, Simon F. Nørrelykke, Markus G. Manz, César Nombela-Arrieta

**Affiliations:** 1 Hematology, University Hospital and University of Zurich, Zurich, 8091 Switzerland; 2 Computer Vision Laboratory, ETH Zurich, Zurich, 8092 Switzerland; 3 Department of Diagnostic and Interventional Radiology, University Hospital Zurich, Zurich, 8091 Switzerland; 4 Department of Microbiology and Immunology, Osaka University, Osaka, 565-0871 Japan; 5 Scientific Centre for Optical and Electron Microscopy–ScopeM, ETH Zurich, Zurich, 8093 Switzerland

**Keywords:** Confocal microscopy, Image processing, Statistical methods, Haematopoiesis, Stem-cell niche

## Abstract

Sinusoidal endothelial cells and mesenchymal CXCL12-abundant reticular cells are principal bone marrow stromal components, which critically modulate haematopoiesis at various levels, including haematopoietic stem cell maintenance. These stromal subsets are thought to be scarce and function via highly specific interactions in anatomically confined niches. Yet, knowledge on their abundance, global distribution and spatial associations remains limited. Using three-dimensional quantitative microscopy we show that sinusoidal endothelial and mesenchymal reticular subsets are remarkably more abundant than estimated by conventional flow cytometry. Moreover, both cell types assemble in topologically complex networks, associate to extracellular matrix and pervade marrow tissues. Through spatial statistical methods we challenge previous models and demonstrate that even in the absence of major specific interaction forces, virtually all tissue-resident cells are invariably in physical contact with, or close proximity to, mesenchymal reticular and sinusoidal endothelial cells. We further show that basic structural features of these stromal components are preserved during ageing.

## Abstract (web summary)

The bone marrow microenvironment modulates haematopoiesis, stem cell maintenance and differentiation. Here, the authors use 3D microscopy to map the topography of haematopoietic stem cell niche stromal components.

## Introduction

Continuous de novo generation of all haematopoietic cell lineages is a major physiological process centralized in bone marrow (BM) tissues during adulthood^1^. High rates of cell production are sustained by haematopoietic stem cells (HSCs), which self-renew while generating a steady flux of multipotent progenitors (MMPs) that mature along the haematopoietic hierarchy^2,3^. Sophisticated mechanisms ensure balanced haematopoietic output and simultaneous HSC maintenance^4,5^. Fundamental regulatory cues are locally delivered by non-haematopoietic BM stromal cells, which include a plethora of increasingly defined endothelial, mesenchymal and neural cell subtypes^6–8^. A well-accepted model of the functional organization of BM posits that stromal cells exert regulation on haematopoietic cells by establishing highly specific interactions in spatially confined microanatomical units, termed niches. These entities favour maintenance or differentiation of distinct haematopoietic subtypes, of which the most widely studied are those governing HSCs^5,9,10^. Therefore, the detailed analysis of the structural organization and interactions established between different BM subsets through in situ microscopy can reveal essential information on the mechanisms that orchestrate haematopoiesis.

Endothelial cells form the linings of BM microvascular networks, which include arterial, transitional (type H) and sinusoidal microvessels^11^. BM endothelial cells are major regulators of haematopoiesis^12–15^. Genetic deletion of crucial haematopoietic factors such as the chemokine CXCL12 or stem cell factor (SCF) in endothelium results in partial to almost complete depletion of HSCs from the BM, thus proving a major functional interplay between these cell types^16–18^. Further supporting this, two- (2D) and three-dimensional (3D) microscopy studies have found that the vast majority of HSCs reside in extraluminal perisinusoidal spaces in direct contact with sinusoidal endothelial cells (SECs)^19–21^. A minor fraction of HSCs localize near arterial and arteriolar structures, which are hypothesized to constitute niches with distinct and alternative functional properties^22,23^. On the basis of spatial and functional data, SECs have also been proposed to embody multiple BM stage-specific niches involved in megakaryocyte maturation and platelet biogenesis, late B-cell development and maintenance of mature circulating B cells, among others^24–28^.

The mesenchymal component of BM stroma has also attracted major interest for its role in regulating haematopoiesis^29^. Mesenchymal progenitor, bone-forming and adipogenic potentials are contained in a subset of fibroblastic reticular LepR^+^ cells, which are most frequently observed in the vicinity of sinusoids^30,31^. The majority of LepR^+^ cells express substantial levels of CXCL12, exhibit the brightest green fluorescent protein (GFP) labelling in *Cxcl12-Gfp* knock-in reporter mice and have thus been termed CXCL12-abundant reticular cells (CARcs)^30,32–34^. Although other mesenchymal stromal cells, which are not equally labelled in CXCL12 reporter mice, have been described to produce CXCL12^35^, CARcs are essential to marrow function as they are additionally major sources of SCF and the pro-lymphoid cytokine interleukin-7^17,30,36^. Thus, CARcs have been successively shown to fulfil principal pleiotropic roles in the regulation of HSCs, multipotent progenitors, lymphoid progenitors as well as natural killer, B and plasmacytoid dendritic cell development^24,36–39^. In these studies, some degree of spatial proximity was observed between all mentioned haematopoietic components and CARcs in BM sections.

Altogether, the cardinal roles of sinusoidal microvessels and CARcs in haematopoietic regulation have been amply demonstrated^9^. Nonetheless, it is currently not known whether these cells exert all reported functions via direct interactions in spatially restricted niches. Conclusions on CARc and SEC interactions have often been drawn merely from data generated from visual identification and manual counting of few events, within limited fields of view and randomly selected tissue regions. However, the significance of the observed spatial arrangements with respect to any proposed interacting partner has, for the most part, not been rigorously tested. Stringent spatial statistical methods to infer interactions between objects require the integration of broad contextual information, such as dimensions and geometry of the tissue volume examined and the absolute number, size and distribution of the entities analysed^40^. These parameters are needed to statistically discern the existence of non-random spatial dependencies indicative of cell–cell interactions within a given 3D anatomical space^41^.

Comprehensive quantifications of BM mononuclear cell (BMMNC) content are conventionally performed via flow cytometry (FC), which is a high-throughput method for rare cell detection in heterogeneous tissues^42^. The application of FC to study stromal cells suggests that despite their functional relevance, both SEC and CARc subsets individually contribute to a minor proportion of total BMMNCs (0.03 and 0.3%, respectively)^10,12,15,30,31,43–45^. However, whether stromal cells are efficiently extracted by tissue disaggregation protocols required for FC remains to be determined. Hence, the usefulness of this technology to quantitatively and qualitatively detect pathology-related alterations in BM stromal composition is unclear. For instance, ageing is accompanied by a functional decline in haematopoiesis, which is partially due to altered HSC function^46^. Age-related disturbances in BM microenvironmental components are hypothesized to contribute to alterations in haematopoietic competence^47^ and include increased adipocyte infiltration^48^, perturbations of the cytokine milieu and reductions in bone volume and type H vessels^15^. Yet, global quantitative and spatial studies of stromal cells within intact BM landscapes and their potential changes during ageing are largely lacking.

The aforementioned questions on BM structure are now becoming addressable by rapidly emerging deep-tissue imaging techniques and automatic bioimage analytical methods^20,21,49–52^. Here, we employed a combination of customized protocols for 3D microscopy, computational tools for image-based quantification and extensively validated spatial statistical methods to analyse the numbers, distribution and spatial coverage of CARcs and sinusoidal endothelial structures in the BM of young and aged mice.

## Results

### Multiscale 3D quantitative microscopy of BM cellular components

We previously employed deep-tissue imaging to study vascular microarchitecture in murine BM femoral cavities^20^. We optimized this methodology to enable the generation of high-resolution 3D reconstructions of BM volumes ranging across multiple scales, from organ-wide to subcellular levels. We have additionally developed a computational pipeline for: (i) automatic detection and segmentation of immunolabelled objects in volumetric datasets, (ii) accurate quantification of cellular populations and analysis of global and/or local spatial distributions and (iii) inference of interaction patterns between defined objects using appropriate statistical analyses.

**Fig. 1:**
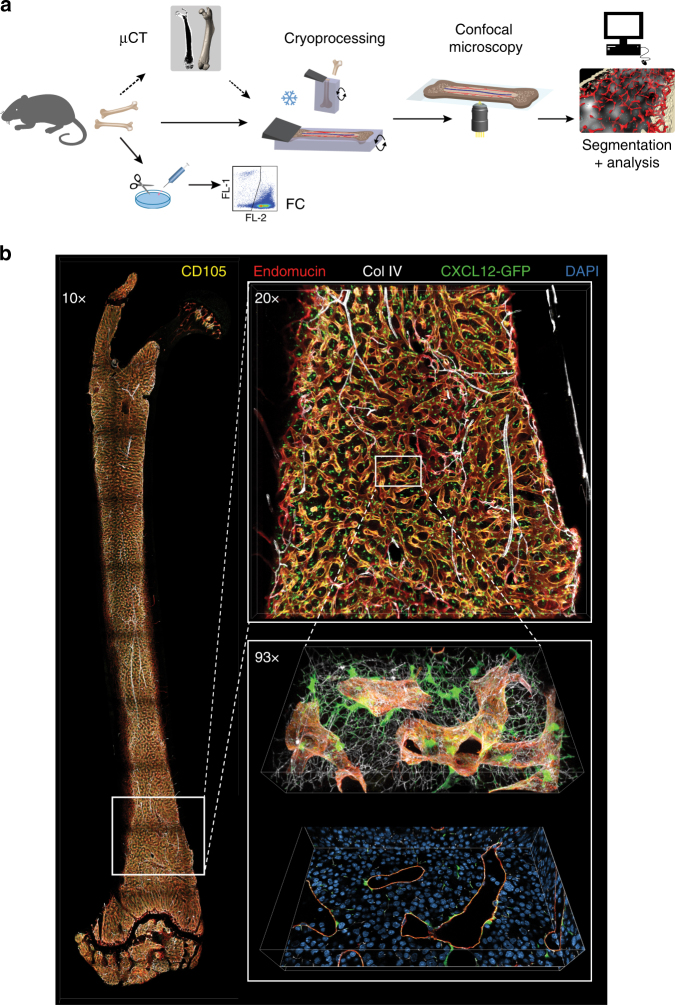
3D quantitative microscopy (3D-QM) of murine BM femoral cavities. **a** Schematic representation of the experimental workflow for preparation, image acquisition and software-based analysis in multiscale imaging of BM tissues. For quantification of total BM volume, femurs are first scanned by µ-CT and then cryopreserved and sectioned for subsequent confocal microscopy. The resulting 3D images are processed and analysed by 3D-QM to quantify mean cellular densities. Cell suspensions from contralateral femurs are prepared and analysed by flow cytometry (FC) when a pairwise comparison of data generated by both methodologies is required. **b** Examples of images acquired at different magnifications covering the entire femoral slice, to subcellular levels of resolution of an immunostained BM from a *Cxcl12-Gfp* transgenic mouse (10×, 20× and 93× magnification). Lower image depicts a single optical section and an orthogonal slice of a confocal image stack in which dimensions in the *z*-axis and details on the nuclear staining with DAPI are visible. See also Supplementary Movie 1

The main steps of the experimental workflow for 3D quantitative microscopy (3D-QM) are summarized in Fig. 1a and extensively described in the Methods section. In brief, optically cleared transversal or longitudinal BM slices are imaged with a confocal microscope at varying resolutions and depths. Initial tissue screening with low optical magnifications allows for fast generation of tissue-scale images that provide a general overview of the global tissue context and enable definition of regions of interest (ROIs), which are subsequently registered at high resolution. Tiled, serial confocal image stacks are assembled as mosaics into single volumetric images (Fig. 1 and Supplementary Movie 1). This multiscale reconstruction approach permits precise visualization of objects ranging from intra- and extracellular macromolecular structures to multicellular networks extending throughout large tissue areas (Fig. 1b).

For image-based quantification we have implemented a combination of commercially available and custom-designed software to segment extracellular, cellular and vascular components of the BM (see Methods). Individual cells are digitally reconstructed as spheres, and continuous networks as surface meshes, which are mapped into rendered volumes (Supplementary Movie 1). Tissue boundaries are defined by applying image-processing operations on the DAPI^+^ fluorescent signal of cell nuclei (Supplementary Fig. 1 and Supplementary Movie 2). Cellular densities of discrete subsets, and spatial trends and dependencies between elements, are assessed using points processes, a validated statistical framework^53^ applied in other research domains and more recently in biomedical image analysis^41,54,55^. The tools employed hereafter for image processing, statistical analysis and data representation have been implemented as user-friendly plugins for Imaris software.

### 3D-QM reveals high abundance and widespread distribution of CARcs in BM

**Fig. 2:**
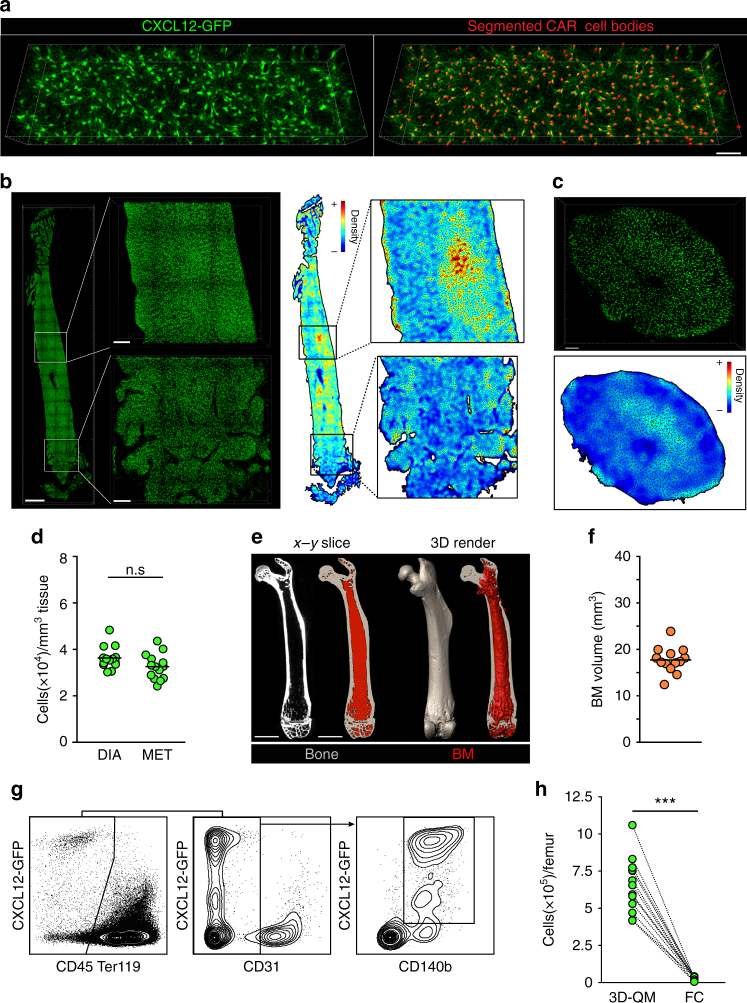
3D-QM reveals significant underestimation of CARcs by FC-based methods. **a** Representative 3D image of a region from the femoral diaphysis showing the typical distribution and morphology of CARcs (left). Annotated image in which all CARc bodies are automatically detected using the Spots module of Imaris software (red spheres). Scale bar, 50 µm. **b** Representative 3D images of CARcs (green) distribution in longitudinal slices of entire BM cavities (left; scale bar, 1 mm) diaphysis (top) and metaphysis (bottom; scale bars, 200 µm). Colour-coded tissue density maps based on the detection and quantification of CARc presence are shown on the right. **c** Representative 3D image of a transversal slice of the femoral bone from a *Cxcl12-Gfp* mouse sectioned at the level of the diaphysis (top; scale bar, 100 µm) and its corresponding tissue density map (bottom) created as in **b**. CARcs are shown in green. **d** Density of CARcs in diaphysis (DIA) and metaphysis (MET) expressed as cells per mm^3^ of BM tissue (*n* = 14). The tissue volume visualized and quantified for CARc presence was 0.17 ± 0.03 µL/femur. **e** Determination of absolute BM volume contained in single femoral bones using µ-CT. Raw images of 2D cross-sections are processed to segment BM (red) and bone (grey; left). Scale bar, 2 mm. A 3D rendering of the entire femur is shown here where the extracted total marrow volume is visible (right). **f** Quantification by µ-CT of BM tissue volumes contained in the femurs from *Cxcl12-Gfp* mice (*n* = 13). **g** Representative FC dot plots and the employed gating strategy for the quantification of CARc frequencies and absolute numbers after enzymatic tissue processing into cell suspensions. **h** Pairwise comparisons of total CARc numbers as enumerated by 3D-QM and FC. Lines connect dots corresponding to contralateral femurs of the same individual mice (*n* = 12). For 3D-QM, individual dots correspond to values obtained from calculating the weighted average of two images from different BM regions. Significance was analysed using the Mann–Whitney test ***P < 0.0001; n.s. not significant with *P* < 0.01. In **d**, **f** bars show the mean and dots represent data from single femurs from different mice

Despite their proven functional relevance, a global quantitative microanatomical analysis of CARcs in BM has not been performed. We employed 3D-QM to analyse the BM of *Cxcl12-Gfp* reporter mice, in which CARcs are tagged by GFP expression^56^. The bright GFP signal was restricted to non-haematopoietic (CD45^-^Ter119^-^), non-endothelial (CD31^-^) cells displaying the typical fibroblastic morphology and marker expression (CD140b) of mesenchymal reticular cells (Fig. 2a and Supplementary Fig. 2a, b). Hence, as previously reported, GFP^bright^ cells in *Cxcl12-Gfp* mice correspond exclusively to CD45^-^Ter119^-^CD31^-^CD140b^+^ CARcs^33^, making this reporter strain the ideal model to specifically visualize and study this cell type via microscopy. Individual CARcs were automatically mapped and their centres of mass detected (Fig. 2a). Direct comparison of software-based segmentation with manual annotation by independent observers confirmed the high precision of automated methods for CARc tracking (Supplementary Fig. 2c and Supplementary Movie 2). Imaging of femoral cavities along the longitudinal and transversal axes revealed no conspicuous regionalization of CARcs in specific BM zones (Fig. 2b, c). Although we could observe local inhomogeneities in tissue maps, CARc density was robustly consistent in different regions when large volumes were analysed (mean total BM 3.51 ± 0.13 × 10^4^ cells/mm^3^, diaphysis 3.78 ± 0.14 × 10^4^ cells/mm^3^, metaphysis 3.21 ± 0.14 × 10^4^ cells/mm^3^) (Fig. 2d).

The widespread distribution and unexpectedly high density of CARcs stands in apparent contradiction with the rare frequencies reported by numerous groups using FC techniques^30,31,43^. To quantitatively assess this discrepancy we employed both methodologies (FC and 3D-QM) in parallel to determine the absolute number of CARcs present in either of the two femoral bones of *Cxcl12-Gfp* mice. FC-based quantification was performed using previously described protocols including tissue enzymatic digestion to facilitate cellular extraction^57^. The values obtained by FC for total BM cell content and frequencies of CARcs in BMMNCs fell within previously published values (Fig. 2g, h)^30,31,43^. For image-based quantification of CARc numbers, femurs were first imaged by micro-computed tomography (µ-CT) and the BM femoral volume was calculated by image segmentation (Fig. 2e, f). Bones were then processed for confocal imaging and global CARc densities were determined in large volumes of metaphysis and diaphysis. Using total organ volume values and mean cellular densities we estimated the absolute numbers of CARcs populating individual femoral bones. Pairwise comparison of the total size of the CARc pool revealed striking discrepancies between 3D-QM and FC (Fig. 2h). In fact, 3D-QM uncovered a total of 6.56 ± 0.53 × 10^5^ CARcs in single femurs, 31.4 ± 4.8-fold greater than those estimated by FC-based quantifications. Therefore, FC failed to detect 95.9 ± 0.58% of CARcs.

### Quantitative analysis of SECs and sinusoidal networks in the BM

**Fig. 3:**
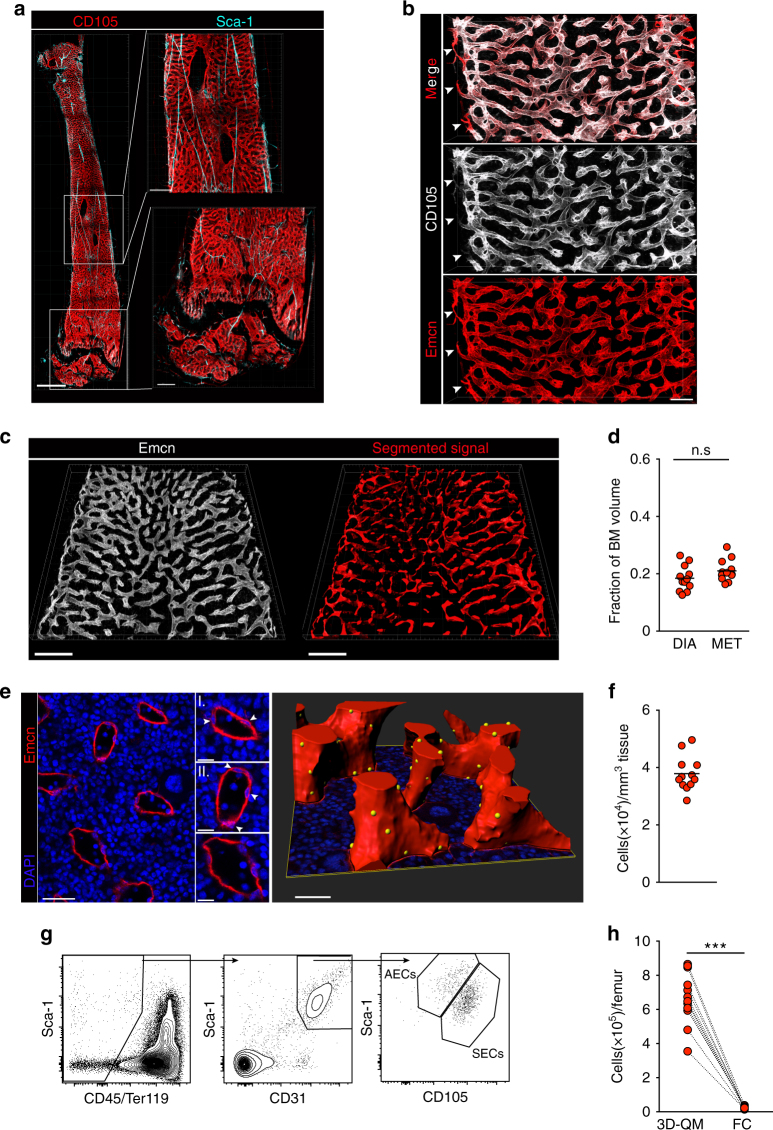
3D-QM analysis of BM sinusoidal endothelial structures. **a** Representative images of the BM microvascular compartment of an entire femoral cavity (left; scale bar 1 mm), including arterial (light blue) and sinusoidal vessels (red). Zoomed-in images (right) reveal distinct features of BM sinusoids and arteries in diaphysis (top) and metaphysis of femurs (bottom). Scale bars, 300 µm. **b** 3D reconstruction of a confocal image stack acquired in a large region of a femoral diaphysis immunostained for the sinusoidal endothelial markers CD105 (white) and Endomucin (Emcn, red). Scale bar, 100 µm. Transitional zone (type H) vessels present mostly at the junction of arterioles and sinusoids in the endosteal regions are strongly positive for Endomucin and weakly express CD105 (marked by arrowheads). **c** Endomucin signal (white) was used as input for our customized algorithm to reconstruct sinusoids in diaphysis (DIA) and metaphysis (MET) of femoral BM cavities. Scale bar 200 µm. See also Supplementary Fig. 4. **d** Fraction of entire BM space occupied by intrasinusoidal volumes in femoral diaphysis and metaphysis (*n* = 11). Bars show the mean and dots represent data from single femurs from different mice. **e** Left panel depicts a representative high-resolution image of a 2D optical section in which the nuclei of SECs are visible and can be manually annotated. Scale bar, 30 µm. Three zoomed-in pictures with examples of detected SEC nuclei (marked by arrows) are shown. Scale bars, 10 µm. The resulting 3D reconstruction of SEC nuclei (yellow) on the segmented sinusoids (red) is shown on the right panel. Scale bar, 30 µm. **f** Quantification of SEC nuclei per mm^3^ of BM tissue volume. Individual dots correspond to values obtained from one single femur by calculating the weighted average of two images from different BM regions (*n* = 12) and bars depict mean values. **g** Gating strategy for identification of BMECs and distinction and quantification of AECs and SECs by FC. **h** Comparison of total SECs per femur as determined by 3D-QM and FC. Lines connect dots corresponding to values determined for contralateral femurs of the same individual mice (*n* = 11). Significance was analysed using Mann–Whitney test. ***P < 0.0001; n.s. not significant with *P* < 0.01

We next focussed on the quantitative characterization of sinusoidal endothelium by 3D-QM. Previous work by us and others showed that arterial and arteriolar vessels feature strong expression of the antigen Sca-1/Ly6a^20,22,23^, which is only weakly expressed by BM SECs (Fig. 3a and Supplementary Fig. 3a). Conversely, SECs express highest levels of CD105 (Endoglin) as well as Endomucin. Both markers display significantly attenuated expression in arterial microvessels (Supplementary Fig. 3a, b). Transitional vessels, which connect arterioles to the nascent sinusoidal network along endosteal surfaces^45^, were also strongly positive for Endomucin while displaying lower CD105 expression (Fig. 3b and Supplementary Fig. 3b). Therefore, differential marker expression enables phenotypic discrimination of BM vascular types by imaging: strong CD105-specific signals delineate sinusoids, Endomucin-specific signals label sinusoids and a minor fraction of transitional vessels, and finally arterial and arteriolar endothelial cells (AECs) are marked by strong expression of Sca-1.

Following this labelling scheme, we used the bright Endomucin-specific signal to determine the density of non-arterial vasculature, and its distribution across the BM. Quantification of the total intravascular volume of sinusoidal networks required the digital reconstruction of entire vessels, which is challenging due to their wide and hollow morphology. In high-resolution images the Endomucin signal was restricted to the contour of sinusoidal walls, while large intravascular spaces appeared as extended patches devoid of fluorescent signal. However, through image processing, vascular lumina demarcated by endothelial-specific signals were filled and continuous tubular networks were rendered (Fig. 3c, Supplementary Fig. 4a, b and Supplementary Movie 3). This analysis confirmed that non-arterial microvessels, mostly sinusoids, uniformly spread throughout BM and collectively amount to a substantial fraction of tissue volume (total BM 20.3 ± 1.2%, diaphysis 19.7 ± 1.3%, metaphysis 21.2 ± 1.0%, Fig. 3d).

In line with our previous results on CARcs, the large extension and volumetric occupancy of sinusoidal vasculature seemed hard to reconcile with previously published extremely low frequencies of ECs in total BMMNCs measured by FC^10,12,17^.Thus, we sought to obtain a realistic estimation of density and absolute numbers of SECs via 3D-QM. BM slices were immunostained for Endomucin and/or CD105, which together with morphological features allowed for a clear discrimination of sinusoidal vessel linings (Fig. 3e and Supplementary Movie 4). Single nucleated SECs were easily detectable through visual inspection and were manually annotated by sequential examination of image stacks. Enumeration of SECs in extended volumes BM from different regions permitted the calculation of SEC densities (Fig. 3f). Based on the homogeneous sinusoidal vascularization throughout marrow and assuming uniform distribution of SECs along microvessel walls, we calculated absolute numbers of SECs in single femurs for which the total BM volume was measured by µ-CT prior to confocal imaging.

Contralateral femurs were analysed by FC for SEC content. We first confirmed that the immunostaining profile employed in microscopy also permitted distinction of SECs from AECs with FC. Indeed, inclusion of antibodies against Sca-1 and CD105 in a typical stromal FC panel segregated the CD45^-^Ter119^-^CD31^hi^ endothelial cell fraction in two distinct subpopulations, namely Sca-1^hi^CD105^int^ cells and Sca-1^int^CD105^hi^ cells, which we reasoned corresponded to AECs and SECs, respectively (Fig. 3g). Consistent with this hypothesis, purified Sca-1^hi^CD105^int^ cells expressed high levels of prototypical specific arterial markers, including *Sox17*^58^, *EphB2* (EphrinB2)^59^ and *Ly6A* (Sca-1*)*^12^, as determined by quantitative reverse-transcription PCR (qRT-PCR; Supplementary Fig. 3c). Concomitantly, cells falling into the Sca-1^int^CD105^hi^ gate were highly enriched in expression of the sinusoidal marker *Flt-4* (vascular endothelial growth factor receptor-3)^12^. In agreement with previous work and with their reported role in recruitment of circulating cells to BM, sorted SECs displayed highest levels of expression of adhesion molecules such as *P-, E-selectin* and *VCAM-1*^60–62^ at messenger RNA and/or protein levels (Supplementary Fig. 3c, d). Both populations expressed prototypical endothelial genes *Pecam1* (CD31), *Tek* (Tie-2) and *Cdh5* (VE-cadherin), further confirming their identity (not shown and Supplementary Fig. 3d). Thus, our dual immunolabelling strategy with CD105 and Sca-1 consistently distinguishes arterial from sinusoidal endothelial subtypes in microscopy and FC, and aligns with previously published protocols for SEC discrimination^44^. Using this approach we determined frequencies and absolute numbers of SECs in femurs of mice in which contralateral bones were analysed by 3D-QM. The values obtained for frequencies of CD45^-^Ter119^-^CD31^hi^ endothelial cells and total BMMNC numbers by FC were consistent with previous reports^12,15,45^. Pairwise comparison with bones analysed by 3D-QM clearly demonstrated that on average 96.6 ± 1.10% of SECs were not detected using current FC methodologies (Fig. 3h). In situ enumeration yielded an estimation of 6.68 ± 0.44 × 10^5^ SECs per femur, which was 33.0 ± 3.4-fold greater than SEC numbers obtained by FC.

Our data proved that the majority of SECs and CARcs are neglected by FC. Yet, it was unclear whether isolation-associated cell losses also affected haematopoietic populations to some extent. We next analysed femoral *Foxp3-Gfp* mice, in which the regulatory T cell (T_regs_) subset is traceable by 3D-QM and FC^63^ (Supplementary Fig. 5a, b and Supplementary Movie 5). We deliberately selected this haematopoietic subset, as the reported frequency of T_regs_ in BM by FC falls in the same range to that of SECs and CARcs (Supplementary Fig. 5b). Quantification of the abundance of T_regs_ in different femurs from the same mice by 3D-QM and FC was not significantly different (Supplementary Fig. 5c, d). These results demonstrate for the first time that FC-based quantifications lead to the selective underestimation of BM stromal cells and thus generate a heavily biased picture of BM composition.

### Continuous CARc networks run along dense ECM fibre scaffolds

**Fig. 4:**
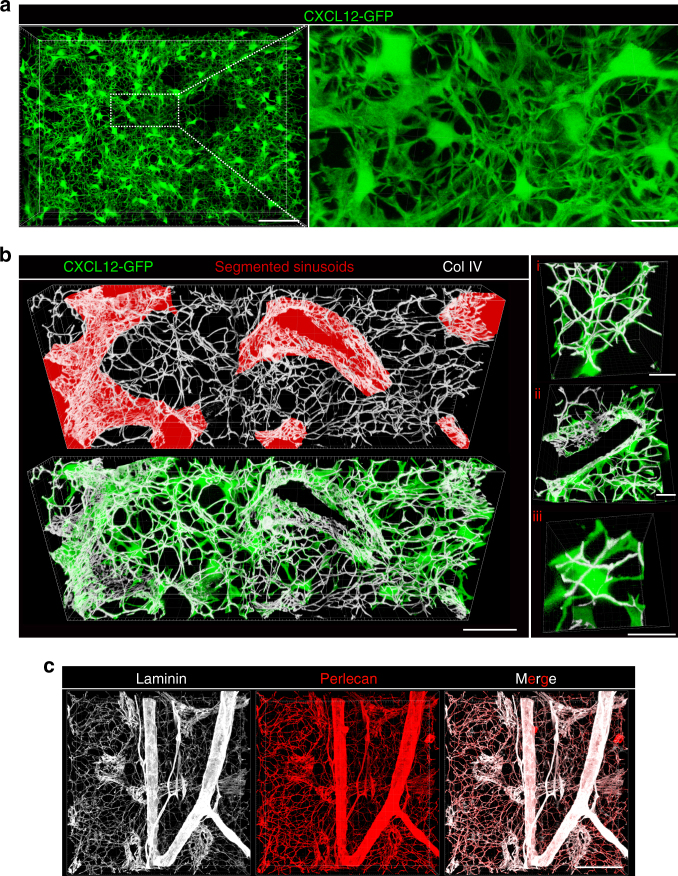
CARcs assemble as dense pervasive networks associated to ECM fibres. **a** 3D high-resolution reconstruction of CARc networks present in femoral BM cavities of *Cxcl12-Gfp* mice. Scale bar, 50 µm. Details of the mesh of CARc cytoplasmic projections connecting the entire network are shown in the zoomed-in image on the right panel. Scale bar, 10 µm. **b** 3D representative image of BM from *Cxcl12-Gfp* mice immunostained for ECM marker (collagen IV-Col IV white) and a sinusoidal marker (Endomucin red). Scale bar, 30 µm. High-magnification images of boxed regions are shown on the right panels. Examples of CARc cytoplasmic projections running along ECM fibres (i), prominent ECM deposition around sinusoidal vessel walls (ii) and a CARc body enwrapped in fine ECM bundles (iii) are depicted. Scale bars, 10 µm. **c** Immunostaining of BM ECM for prototypical markers Laminin and Perlecan showing prominent ECM deposition in arterial walls. Scale bar, 50 µm

Up to this point, our image-based analysis of CARcs focussed in the visualization of cellular somata, which permitted localization and quantification of cellular centroids. Nonetheless, enhanced resolution imaging resolved unappreciated subcellular details of CARcs. Individual cytoplasmic projections emitted from the somal perimeter became visible uncovering an unanticipated degree of 3D interconnectivity in the network (Fig. 4a). Indeed, CARcs form a lattice in which all elements are physically adjoined via extension of numerous, elongated and thin processes. Individual pseudopodia followed complex trajectories and branched into multiple ramifications, which made it challenging to distinguish boundaries between neighbouring CARcs. The CARc continuum projected throughout the entire extravascular space, with only very limited regions remaining unreached by cellular extensions.

Morphologically, the CARc infrastructure evokes those formed by fibroblastic meshworks of lymphoid organs, known to produce and assemble around extracellular matrix (ECM) fibres^64,65^. Although the ECM has been proposed to participate in the microenvironmental regulation of haematopoiesis and tissue remodelling, the distribution and heterogeneity of ECM components are poorly understood^51,66,67^. Using immunostaining of typical ECM proteins, we could visualize the microarchitecture of ECM microfilaments and their coverage of different BM regions (Fig. 4b). In a preliminary analysis we did not detect major heterogeneities in the regional distribution of the ECM components Collagen IV, Laminin, Perlecan and Fibronectin (Fig. 4c and not shown). Major deposition of ECM was found in the abluminal surfaces of sinusoids (Fig. 4b, ii) and, to a greater extent in arterial walls (Fig. 4c). ECM fibres projected from endothelial surfaces into the extravascular space. The ECM web-like infrastructure was tightly associated with the multitude of CARc projections, which strictly followed the trajectories of ECM fibres and split along their multiple branching sites. CARc bodies were also partially enwrapped by thin ECM bundles (Fig. 4b, iii and Supplementary Movie 6). Thus, ECM fibres and CARcs associate into a proteo-cellular scaffold, which constitutes the core infrastructure of BM tissues.

### The vast majority of the BM cellular content spatially associates to CARcs and SECs

**Fig. 5:**
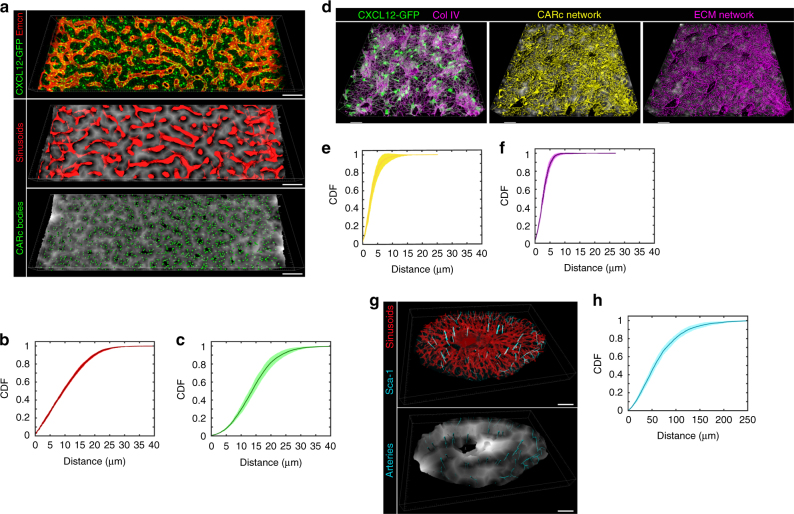
Spatial coverage of BM spaces by SECs and CARc networks. **a** Representative examples of BM reconstructions from confocal image stacks (top) employed to automatically segment sinusoids (middle) and CARc bodies (bottom). Scale bar, 100 µm. The 3D ESD depicts in grey scale the distance from every voxel in extravascular spaces to the closest voxel containing either SECs or CARcs. **b**, **c** CDF of ESD to the closest (**b**) sinusoid (*n* = 6) and (**c**) CARc soma (*n* = 6). **d** High-resolution 3D reconstructions employed to visualize and segment entire CARc networks (cellular bodies and projections) and ECM fibres stained with Collagen IV (Col IV). 3D ESD (grey scale) to segmented CARcs (middle) and ECM (right) networks. Scale bars, 30 µm. **e**, **f** Cumulative distribution functions of the ESD to CARcs (*n* = 7) (**e**) and ECM (*n* = 7) (**f**) networks. **g** 3D image of a transversal femoral section depicting Sca-1 signal and segmented sinusoids (top). In the bottom panel the arterial Sca-1 signal is segmented and the ESD to arteries is shown in grey scale within the same image. **h** CDF of the ESD to arteries/arterioles (*n* = 5 bones in which data from metaphysis and diaphysis are combined). In **b**, **c**, **e**, **f**, **h**, the mean accumulated frequencies at each distance are represented by solid lines, with the standard deviations shown as the envelope

Our observations on the abundance and topographical extension of SECs and CARc networks point to a much wider physiological and spatial influence than previously assumed. To quantitatively evaluate how the BM tissue landscape relates to these stromal cells we performed detailed spatial analyses. Sinusoids were segmented and CARc centroids identified and represented as spheres. The distance of each voxel in the BM extravascular space to the closest anatomical landmark of interest was automatically measured and represented as a 3D distance map in a grey scale (Fig. 5a). We calculated the cumulative distribution function (CDF) of this empty space distance (ESD) transform, which represents the accumulated distribution of distances that any tissue-resident cell may adopt with respect to stromal objects studied. Figure 5b, c depicts the CDF of the ESD to sinusoids and CARcs computed in multiple images spanning large BM volumes. Notably, within the native context of the BM, 52.9 ± 0.9% or 29.6 ± 1.8% of locations are within 10 µm of the nearest sinusoid or CARc body, respectively. More importantly, almost the entire BM space was contained within less than 30 µm of the closest sinusoid (99.4 ± 0.2%) and the surface of the nearest CARc soma (97.6 ± 0.5%, Fig. 5a–c). However, this analysis considered only CARc bodies as potentially interacting structures, while excluding the numerous ECM-associated CARc projections, which are most likely active platforms for cellular crosstalk. We next performed similar spatial analyses in high-resolution images in which subcellular reticular projections of the CARc network were resolved and segmented together with the ECM network. Analysis of similar ESD transforms revealed that the entire BM space is contained within less than 7 µm of the closest CARc surface and adjacent ECM fibre (Fig. 5d–f). In line with previous reports^21^, arterial networks were significantly less abundant. Given the heterogeneous presence and longitudinal trajectories of arterial vessels, we analysed transversal slices of metaphysis and diaphysis to best capture their spatial influence (Fig. 5g). Quantitative analysis revealed that only 5.6 ± 0.5% of the BM extravascular volume is periarterial/arteriolar (≤10 µm) and a minor fraction (23.4 ± 1.7%) is contained within 30 µm (Fig. 5h). Therefore, ubiquitous ECM and CARc networks are physically juxtaposed to virtually all BM-resident cells, sinusoids are pervasive niches and arteriolar networks form highly restricted spatial domains in the BM microenvironment.

### CARcs significantly accumulate in perisinusoidal regions

**Fig. 6:**
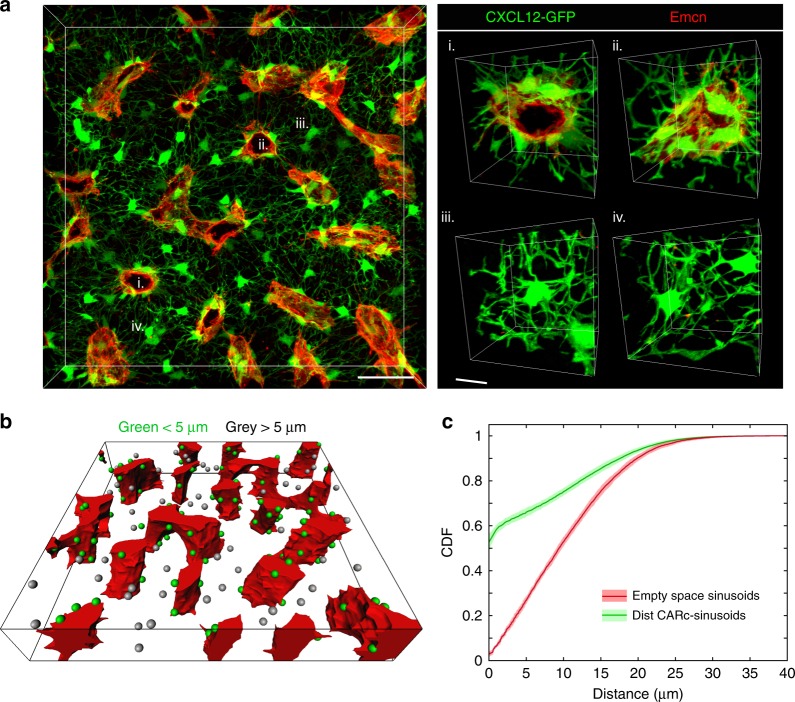
CARcs accumulate in close physical contact with sinusoidal vessel walls. **a** Representative 3D image of BM tissues depicting immunostained sinusoidal vessels (red) and GFP^+^ CARcs (left). Scale bar, 50 µm. Right panels show higher magnification images of perisinusoidal clusters of CARcs (top) and examples of large, non-perivascular CARcs (bottom) Scale bar, 10 µm. **b** Rotated 3D view of a rendered volume from the segmented image in **a**, in which CARc subsets are classified and colour coded according to distance to nearest sinusoid. From the quantitative analysis, two populations of perisinusoidal and non-perisinusoidal are evident (non-perisinusoidal > 5 µm distance from CARc surface to closest sinusoidal surface). **c** Side-by-side comparison of the CDF of the distance to nearest sinusoid evaluated at all positions, as well as evaluated at CARc centroids. Solid lines represent mean distance and envelopes indicate standard deviations. Graphs correspond to the pooled data from a total of 3 different regions per bone and 6 different bones. Statistical significance was analysed using two-sample Kolmogorov–Smirnov and *P* < 10^−42^ for all samples

Confirming previous reports^30,35^, we frequently observed CARcs in direct contact with sinusoids (Fig. 6a), for which both stromal components have been proposed to cooperate in a functional unit^5^. Nonetheless, that CARcs are spatially associated to sinusoids has not been formally shown and based on our data any cell type could be mistakenly regarded as perisinusoidal. We therefore decided to statistically test the existence of spatial dependencies between both stromal components. Sinusoidal vessels and CARcs were mapped in 3D images and the CDF of the distances to nearest sinusoids evaluated at all extravascular locations was compared to that evaluated at the spatial coordinates of all CARc centroids. This analysis demonstrated a highly significant accumulation of CARc bodies in direct adjacency to sinusoidal vessel walls (Fig. 6c). Interestingly, the distribution of distances of CARcs to sinusoids was strongly conserved between different regions and bones investigated, suggesting a robust mechanism underlying these interactions. The fraction of CARcs in direct contact with sinusoids was 64.0 ± 0.7%. We observed the presence of clusters of perisinusoidal CARcs displaying a flattened morphology and forming an almost continuous layer along the endothelial walls (Fig. 6a). Nonetheless, visual inspection of the CDF of CARc distribution further suggested the existence of an additional non-perivascular subpopulation, which could be easily discriminated by classification of CARcs with a predefined threshold of the distance to sinusoids (<5 µm distance from cell centroid to nearest sinusoidal surface) (Fig. 6a–c and Supplementary Movie 7). Of note, non-perisinusoidal CARcs exhibited a microglial-like morphology and seemed to act as interconnecting nodes between distant perisinusoidal cells through emission of numerous radial projections. Thus, CARcs collectively display a non-random spatial affinity towards sinusoidal vessels, which is indicative of a functional interaction between both components. Nonetheless, a subpopulation of CARcs is consistently distinguished based on its non-perivascular localization.

### CARc and SEC networks are preserved in homeostatic ageing

**Fig. 7:**
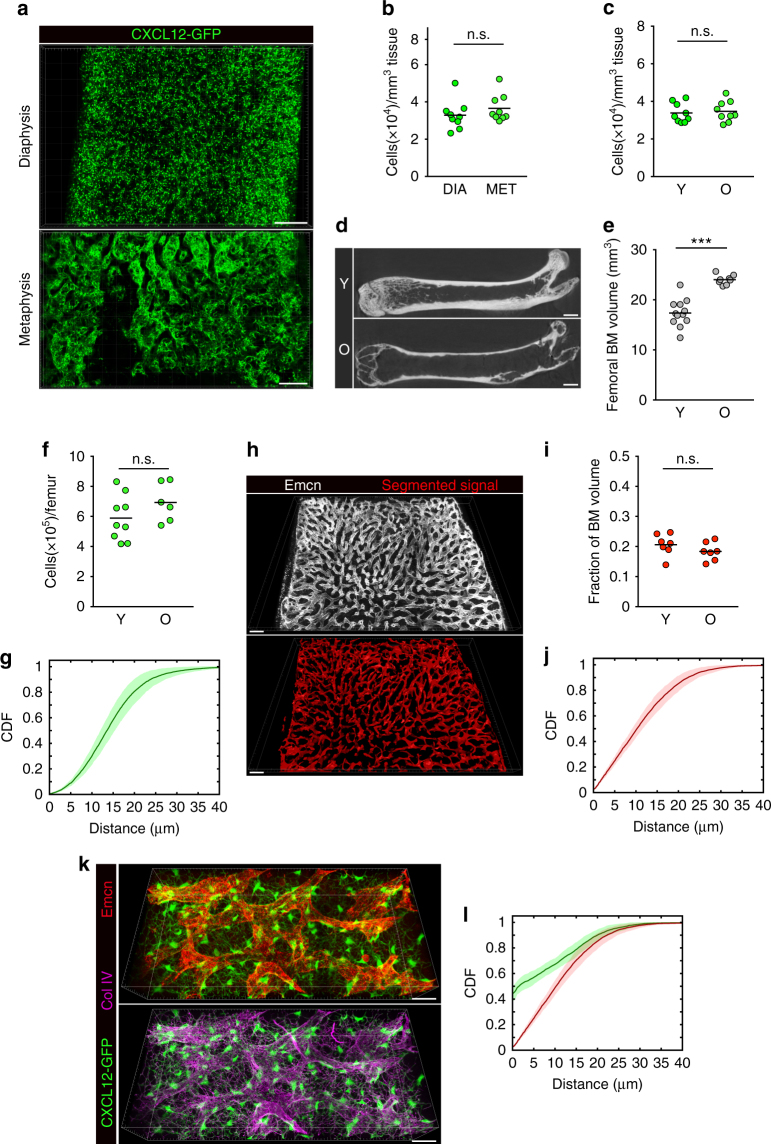
Analysis of CARc and sinusoidal vessel networks in ageing. **a**, **b** Representative 3D images and quantification of CARc densities in the femoral metaphysis and diaphysis of old (O) mice. Scale bars, 200 µm. **c** Mean CARc densities in femoral BM of young (Y, 2–3 m/o) and old (O, 20–24m/o) mice. **b**, **c** Bars show the mean and dots represent individual values for single femurs from different mice (*n* = 9 per group). **d**, **e** Representative µ-CT images (orthogonal projection corresponding to a 200 µm thickness; scale bar, 1 mm) and quantification of total BM volume of Y and O mice (***P < 0.0001). Bars show the mean and dots represent individual values for single femurs from different mice (*n* = 12 per group). **f** Absolute numbers of CARcs in single femurs of Y and O mice. **h** Confocal image and segmented reconstruction of the Emcn signal in BM of aged mice. Scale bars, 100 µm. **i** Fraction of BM volume occupied by sinusoids in femurs from Y and O mice. Bars show the mean and dots represent individual values for different femurs (*n* = 7 per group) (n.s. not significant with *P* < 0.01). **g**–**j** CDF of the ESD to CARcs (**g**) and sinusoidal vessels (**j**) in aged BM. **k** High-resolution image of CARc networks, ECM and sinusoidal vessels in femoral BM from one aged mouse. Scale bar, 20 µm. **l** CDF of the distance to nearest sinusoid evaluated at all positions (red), as well as evaluated at CARc centroids (green) in BM from old mice. Solid lines represent mean distance and envelopes indicate standard deviations. Statistical significance was analysed using two-sample Kolmogorov–Smirnov and *P* < 10^−21^ for all samples. Data in **g**, **j**, **l** correspond to images of large BM regions of metaphysis and diaphysis of femoral bones from seven different mice

We next sought to understand whether changes in quantitative and structural features of SEC and CARc networks arise in old mice as a consequence of ageing. We employed 3D-QM to compare femoral BM cavities of cohorts of young (2–3 months old) and old (20–24 months old) mice. Unexpectedly, quantification of CARc bodies revealed no major differences in old mice as compared to young counterparts (Fig. 7a–c). As previously reported, in certain aged mice we found prominent infiltration of adipocytes concentrated in the distal metaphysis of femurs, which resulted in partial perturbation of the overall stromal parenchyma (not shown). Nonetheless, the mean density of GFP^+^ cell bodies in these regions was not significantly reduced and was thus comparable to that of lean diaphyses analysed (Fig. 7b). Of note, the absolute numbers of CARcs in femurs of aged mice were slightly increased compared to young mice, likely due to the augmented volume of femoral cavities as a consequence of the gradual loss of trabecular and cortical bone density associated to ageing (Fig. 7d–f). Similar to CARcs, the intravascular volume of sinusoids did not vary significantly in BM from old mice (Fig. 7h, i). Furthermore, the distribution and overall coverage of BM spaces by CARcs and sinusoids remained unchanged, as reflected by the similar CDF of the ESDs with respect to these structures in young and aged mice (Fig. 7g, j compared to 5b, c). Although we did observe a modest decrease in the fraction of perisinusoidal CARcs in old mice, the association of CARcs with ECM and their spatial arrangement with respect to sinusoids were mostly preserved during ageing (Fig. 7k, l). Altogether, variability was substantially higher in all parameters measured in the BM of old mice, but with our analytical tools no major age-related perturbations in the structural features of the stromal networks were apparent.

## Discussion

Multicellular organ function is the net effect of the concerted interplay of tissue constituents in space and time. Essential to our understanding of BM physiology is: (i) the identification, classification and quantification of the cellular subsets present in BM, and (ii) detailed knowledge on their structural organization into an integrated tissue architecture, which ultimately defines the nature and frequency of the interactions established. Here, we exploit the unique benefits of 3D-QM to unveil fundamental aspects of the BM microenvironment. We demonstrate that standard FC methods for cellular analyses fail to capture the vast majority of BM SECs and CARcs, which are strikingly abundant. Systematic comparison of FC with multimodal imaging data reveals that either the cellular isolation procedures or the analytical process of FC entail a disproportionate loss of stromal cells. Although unclear from our studies, inefficient isolation may be explained by the fact that stromal cells form compact, sessile networks relying in firm adherent interactions, which are likely resistant to digestion and extraction into single cell suspensions.

FC-based comprehensive population analyses have been historically central to the dissection of the haematopoietic hierarchy^42^. However, FC relies on the premise of nearly complete recovery or homogeneous loss of distinct cell types during isolation procedures. Our results are evidently at odds with this assumption in what pertains stromal cell isolation, and further raise fundamental questions. First, whether inhomogeneities in isolation yields also apply to haematopoietic subsets needs to be thoroughly assessed, especially for cells exhibiting elongated shapes and strong adhesive phenotypes such as those of the histiocytic and antigen presenting fractions. Second, it will be crucial to understand to what extent the stromal populations extracted into suspensions and extensively studied at functional and/or transcriptomic levels ex vivo are representative of the in vivo cellular pool. Indeed, the existence of certain subgroups of stromal cells completely intolerant to isolation protocols may have been fully missed up to now. Finally, biases in cell isolation may not only arise from cell-specific but also tissue-dependent factors. In recent work, Steinert et al.^68^ demonstrated through meticulous image-based quantification that recovery of memory CD8^+^ T cells dramatically varied between organs, possibly due to the divergent biophysical properties of tissues. By providing a realistic account of memory T-cell numbers and anatomical distribution, this study led to a radical revision of prevalent models of immunological memory^68^. Biomechanical features of tissues may substantially change with alterations of structure, cellular and molecular composition driven by pathological stimuli. Thus, the dynamic variations in the BM stromal makeup found to occur during haematologic neoplasias, chemotherapeutic interventions or infections should be interpreted with caution until ascertained via in situ imaging^69–71^. We exploit this approach to reveal that key quantitative and spatial features of CARc networks and sinusoids are unexpectedly robust and maintained during homeostatic ageing. In contrast to the reported age-induced loss of type H vessels^15^, sinusoidal structures remain largely intact in the different BM regions and intrasinusoidal volumes were unchanged. Similarly, the numbers of CARcs and their spatial relationship to sinusoids do not vary significantly in aged BM. Future analyses should determine whether and to what extent the age-induced perturbations of HSC function and haematopoiesis^47^ relate to spatial and structural changes in BM organization.

According to image-based estimations, and presuming only minor losses in haematopoietic cell recovery, the frequency of CARc and SEC populations is approximately 30-fold higher than assumed. Such a substantial upward revision of abundance has major implications on the perceived functional relevance of stromal cells and their proposed mode of action. Hitherto regarded as specific elements of confined niches, we demonstrate that CARcs and SECs are spatially dominant and available for physical contact with virtually all, or the majority of cellular elements in BM. Consequently, even in the absence of real interaction forces, the expected levels of association between these stromal cells and any given BM cell type will be very high. In light of our results, the significance of the reported proximities between certain haematopoietic subsets and CARcs or sinusoids could be potentially explained by pure chance. Nevertheless, as currently defined, CARcs and SECs are most likely heterogeneous mixtures of cell subtypes. A deeper understanding of such heterogeneity could help uncover zonation and labour division within these stromal pools, which would explain their multiplicity of functions, and potentially reveal the existence of highly specific stromal–haematopoietic interactions. We anticipate that 3D-QM will be pivotal in the detection of subcategories of stromal cells, based not only on phenotypic and functional criteria but also on spatial traits. Our data strongly point to the existence of two subpopulations within CARcs, based primarily on direct adjacency to sinusoidal surfaces and morphological features. Further studies should help to understand whether these two fractions represent sublineages with divergent roles.

The pipeline presented herein is widely applicable for the dissection of microanatomical features and interrogation of spatial interactions within biological tissues. The power of our approach is strongly based on the use of tissue processing methods that absolutely respect the antigenicity and structural integrity of highly sensitive subcellular structures, thereby enabling their visualization with unprecedented minute detail. We generate 3D reconstructions of intact infrastructures made of ECM fibres and CARc projections, which ultimately underlie the massive spatial influence of these cells. Furthermore, careful treatment and analysis of image datasets is essential. This includes the evaluation of our segmentation strategies through comparison to a manually annotated ground truth, or visual inspection of multiple random regions. Importantly, we propose for the first time the use of points processes, a well-established toolbox of spatial statistics, to study spatial relationships in BM^41,53^. Multiple groups have previously investigated potential spatial dependencies (mostly between HSCs and stromal or haematopoietic subsets) through comparison of empirically observed spatial data to that generated through simulations of randomly distributed spots in 2D and 3D microscopy images^21,22,36,52,72–74^. When performed in realistic, high-quality tissue images, the conclusions derived from these approaches with regard to the levels of random spatial associations of sinusoids and CARcs are well in line with ours^21^. Nonetheless, the criteria used to define randomness as well as the statistical methods for testing significant differences in spatial distributions vary largely, have not always been ideal and thus can lead to inaccurate conclusions on potential interactions. The use of the available ESD as a benchmark for complete spatial randomness avoids the need for additional a priori assumptions required to generate simulations, and provides comprehensive information on the anatomical constraints in which the observed spatial associations take place.

In summary, we show the power of combining quantitative imaging techniques and spatial statistics to reveal novel and key information on the cellular composition, functional interactions and homeostatic regulation of haematopoietic tissues. The datasets presented here can be further used as structural blueprints of stromal network organization and spatial coverage of the BM space in homoeostasis, which will serve to explore and identify novel phenotypes associated to haematological diseases.

## Methods

### Mice

C57BL/6J were purchased from Charlesriver. *Cxcl12-Gfp* mice have been previously described^34^ and *Foxp3-Gfp* knock-in mice^63^ were provided by Nicole Joller (University of Zurich, Institute of Experimental Immunology). All mice were analysed at 12–20 weeks of age. For ageing experiments, young mice were 8–12 weeks old and aged mice 20–24 months old, which were selected for displaying no apparent signs of health impairment at the moment of euthanasia. Experimental animals were not randomized and experiments were performed in a non-blinded fashion. Animals were maintained at the animal facility of the University Hospital Zürich and treated in accordance with the guidelines of the Swiss Federal Veterinary Office. Experiments and procedures were approved by the Veterinäramt des Kantons Zürich, Switzerland.

### Reagents and antibodies

A detailed list of all the reagents employed in this study, including antibodies for FC and 3D-QM and the concentrations at which they were used, is provided in Supplementary Table 1.

### Flow cytometry analysis and cell sorting

For BM cell analyses, murine bones were first harvested and cleaned thoroughly. To ensure precise quantification, femurs were consistently dissected below the hipbone and above the kneecap. BM content was flushed directly into 5 mL of medium (Dulbecco's modified Eagle's medium GlutaMAX™, 10 mM HEPES, 10% foetal bovine serum) using a syringe. Remaining pieces of bones were carefully minced into small-sized fragments (1 mm) and mixed with flushed BM content. Tissue suspensions were thoroughly homogenized by gentle and repeated mixing using a 1 mL pipette to facilitate dissociation of cellular aggregates. Enzymatic digestion was performed by incubation in DNAse (0.2 mg/mL) and Collagenase Type 2 (0.04 g/mL) at 37 °C for 45 min under gentle rocking. Resulting cell suspensions were then filtered through a 70 µm cell strainer, washed in phosphate-buffered saline (PBS), blocked for 15 min using TruStain fcX™ and successively stained with cocktails of fluorescently labelled antibodies for 30 min at 4 °C. Cells were then washed twice with PBS, resuspended in PBS containing 4',6-diamidino-2-phenylindole (DAPI; 0.5 µg/mL) and analysed on a LSR II Fortessa (BD Biosciences). Data analysis was performed using the FlowJo 10 software package.

### RNA isolation and quantitative PCR

BM cell suspensions were prepared and immunostained as for FC analysis described above. Cellular populations of interest were sorted using a FACS Aria (BD Biosciences) equipped with 4 lasers. A total of 5000 to 10,000 cells of each endothelial subtype were directly collected in RLT lysis buffer (Qiagen). Genomic DNA (gDNA) was depleted from cellular lysates using gDNA eliminator columns (Qiagen), RNA was extracted using the RNeasy Plus Micro Kit (Qiagen) and complementary DNA (cDNA) was generated with the High-Capacity cDNA Reverse-Transcription Kit (Thermo Scientific), following the manufacturers’ instructions. Gene expression was measured with a 7500 Fast Real-Time PCR System using either TaqMan® probes or Power SYBR® Green. A full list of primer pairs employed for individual gene detection can be found in Supplementary Table 2.

### BM slice preparation, immunostaining and optical clearing

Methods for 3D imaging of BM were adapted from previously published protocols^20^. Mouse femurs were isolated, cleaned and immersed in PBS/2% paraformaldehyde for 6 h at 4 °C, followed by a dehydration step in 30% sucrose for 72 h at 4 °C. Femurs were then embedded in cryopreserving medium (optimal cutting temperature (OCT)) and snap frozen in liquid nitrogen. Bone specimens were iteratively sectioned using a cryostat until the BM cavity was fully exposed along the longitudinal axis. The OCT block containing the bone was then reversed and the procedure was repeated on the opposite face until a thick bone slice with bilaterally and evenly exposed BM content was obtained. For transversal slices, bones were manually cut after fixation with a razor blade along the transversal axis in 3–4 fragments, which were frozen separately. The procedure then continued as described above for longitudinal sections. Once BM slices were generated, the remaining OCT medium was removed by incubation and washing of the bone slices in PBS 3 times for 5 min. For immunostaining slices were incubated in blocking solution (0.2% Triton X-100, 1% bovine serum albumin (BSA), 10% donkey serum in PBS) overnight at 4 °C. Primary antibody incubations were performed in blocking solution for 3 days at 4 °C, followed by overnight washing in PBS. Secondary antibody stainings were performed for another 3 days at 4 °C in blocking solution but in the absence of BSA to avoid cross-absorption. Immunostained thick femoral slices were successively washed in PBS overnight and incubated in RapiClear 1.52, for a minimum of 6 h, which typically increased imaging depth to 150 µm from the tissue surface without significant loss of signal intensity. The clearing protocol employed is fast, compatible with all fluorescent probes and proteins tested, and preserves the integrity of subcellular structures.

### Confocal imaging

For observation under the confocal microscope, BM slices were mounted on glass slides using vacuum grease. Confocal microscopy was performed with 10× (HCX PL FLUOTAR), 20× (HC PL APO CS2), 63x (HCX PL APO CS2) and 93× objectives (HC PL APO motCORR) on SP5 or SP8 Leica confocal microscopes. Low-magnification tissue-wide images were generated and ROIs were defined for subsequent detailed acquisition with 20×, 63× and 93× objectives. Image analysis was exclusively performed in high-resolution images, which met quality standards. The specific ranges for the acquisition parameters for each type of objective are provided in Supplementary Table 3. Depending on the microscope, a set of monochromatic lasers (405, 488 nm, 561 and 633 nm) or a white light laser (470–670 nm) tuned to specific wavelengths was employed. Combinations of up to five fluorescent dyes (DAPI, AF488, Cy3, AF594 and AF647/AF680) were simultaneously used in our experiments. Detection filters were set to match the spectral properties of fluorochromes, and single dye controls were introduced when necessary to confirm lack of signal spillover between channels. Fluorescence was detected using ultrasensitive Leica Hybrid detectors (HyD). Attenuation of fluorescent signal with tissue depth was minimized using the tool for laser intensity correction along the *z*-axis available on the Leica Application Suite X. Stitching of confocal image stacks was also performed in the dedicated tool included in the image acquisition/analysis software.

### Micro-computed (µ-CT) tomography of femoral bones

To quantify the total BM volume in the femoral cavity µ-CT images of pre-fixed entire mouse femurs were acquired with a Bruker SkyScan 1176 in spiral scan mode and cone beam. Scans were performed with an X-ray peak voltage of 50 kV at a dynamically modulated current, typically around 270 µA. We used a 0.5 mm aluminium filter for beam energy spectrum modulation. The resulting images, reconstructed with the vendor-specific software, had an isotropic resolution of 8.67 μm/voxel. After acquisition, femurs were frozen and processed for 3D confocal imaging.

### Image processing and segmentation of BM components

Confocal image stacks were rendered into 3D volumes and analysed using Imaris v8.2 (Bitplane AG) and MATLAB software version 9.1 (The Mathworks, Inc.). The Imaris Programming Interface was used to implement all the MATLAB-generated image-processing methods described herein, as plugins for Imaris (ImarisXT). When large regions were imaged, the different tiles were stitched with the Leica Application Suite (LAS) X software (Leica microsystems). Image segmentation methods employed in this study made use of the image intensities to classify voxels and determine their correspondence to segmented BM components, which then allowed for digital reconstruction of cells or networks as objects in 3D space. Object volumes were subsequently calculated by multiplying the total number of voxels they occupied by the voxel volume calculated separately for each image stack and acquisition settings. The precise methods for segmenting individual BM components analysed in this study are described in detail below:

Segmentation of DAPI fluorescent signal for determination of absolute tissue volumes: to determine tissue volumes contained in the ROIs, the outer boundaries of BM tissues were defined by image processing of the DAPI signal (Supplementary Fig. 1). A Gaussian filter was employed to smoothen the DAPI signal in regions with compact cellular content, which were then segmented by thresholding. Subsequently, masks were subjected to opening followed by closing operations to separate and merge regions, respectively. A circular morphological operator was used for this operation with a variable radius that defined the size of the regions to be opened/closed. As a final step, connected components in the resulting binary mask were classified slice-wise by their area. Small non-segmented areas were considered voids between cells and included in the mask, whereas limited segmented regions disconnected from the body of the tissue were considered noise and excluded from the mask. The result was a 3D binary image that excludes empty volume as well as bony structures.

CAR cells: CARcs were detected and their spatial coordinates automatically annotated by using the Imaris Spots utility, which finds spherical objects with a predefined radius (3.75 µm in the case of CARcs). The goodness of this automatic algorithm employed for CARc detection was evaluated in three manually annotated images with a total of 182 ± 3.74 annotated cells per image considered as ground truth (GT). Manual annotation was performed in Imaris by three different researchers independently to account for inter-observer variability. For this evaluation, each detected cell was assigned to its closest sphere in the GT (such pair is called corresponding spheres). Detected spheres were counted as true positive (TP) when the distance between a corresponding pair was smaller than 5 µm. Otherwise it was counted as false positive (FP). All GT spheres without assigned corresponding pair were considered false negatives (FN). Precision and recall were then calculated as follows:

$${\mathrm{{Precision}}} = \frac{{\mathrm{{TP}}}}{{\mathrm{{TP}} + {\mathrm{{FP}}}}}\;\;{\mathrm{{Recall}}} = \frac{{\mathrm{{TP}}}}{{\mathrm{{TP}} + {\mathrm{{FN}}}}}.$$

Mean and standard deviation were calculated between the results of the different individuals. Images were discarded when the staining quality did not allow for automatic detection with the precision and recall reported here.

Segmentation of CARc networks, ECM and arterial vessels: network-like structures were segmented using the *Surface* segmentation utility available in Imaris, which detects objects based on local intensities. The resulting segmentation was visually inspected to remove small individual segmented objects components of high sphericity, which were regarded as noise.

Sinusoidal vessel segmentation: in high-resolution 3D images fluorescent signals corresponding to sinusoidal markers could only be observed along the cell surface of SECs in the endothelial walls, while the lumina and extravascular regions displayed a low to background signal intensity. In order to generate full solid reconstructions of vascular volumes we implemented methods to fill vessels as illustrated in Supplementary Fig. 4. Images were padded with their reflection at all borders to minimize problematic border effects in the different image processing steps. Local thresholding was initially applied and thereafter a series of morphological image processing operations were implemented. The first set of morphological operations consisted of closing (to homogenize foreground pixels in proximity), opening (to remove sparkle noise) and once more closing (to generate continuous vessel walls) with a circular morphological operator of variable radius. A selective region filling method was then applied to fill the vessel lumen on each individual optical section. Finally, 3D closing was used on rendered images to avoid discrepancies between contiguous slices generated by small discontinuities in the signal. We checked that stitching artefacts did not affect the segmentation quality. Special attention had to be placed in regions where vessels were close together, since a wrong choice of segmentation parameters could lead to over-segmentation of the space between them. Our quality criterion for the selection of parameters was that the intravascular space was completely reconstructed. We qualitatively evaluated the precision of this segmentation method in randomly selected 2D confocal optical sections of all samples used (Supplementary Fig 4c).

Segmentation of total BM volume in µ-CT-generated images: images were processed to first segment the whole femur using the same approach as described above for sinusoidal vessel segmentation. The resulting volume was subsequently thresholded to create two masks: one for the BM content and the other for the voxels corresponding to bone. The volume of these structures was directly calculated from the mask.

We exclusively analysed BM slices in which integrity of the entire bone marrow cavity or at least of the complete metaphysis or diaphysis was optimal and met quality standards for spatial analysis.

### Spatial statistics and analysis of cellular interactions

The representation of BM components as segmented objects allowed us to study cellular subsets as a point pattern (*x*), which was observed within a defined region (*W*) corresponding to extravascular regions of BM tissues. For this analysis, the extravascular compartment was defined as the intersection of the DAPI mask and the extravascular volume. Thus, the population of cells detected was defined as *x∩W*. This representation permitted the use of spatial statistic methods, which were implemented in MATLAB as plugins for Imaris (requiring ImarisXT module). The ESD in *W* (denoted *Z*) was used as a descriptor of the way in which 3D space is constrained by different cellular structures. For a given cellular structure, this function assigns each voxel (*u*) a distance to the closest structure as *Z(u)*. We used the Euclidean distance, with distance zero set at every voxel that contains at least a fragment of the surface of the given structure. Voxels corresponding to the inside of the studied structures were excluded. Intravascular regions were also excluded so that only extravascular tissue regions were considered. To study the distribution of ESDs with respect to different structures, a histogram of distances for all voxels was formed and then summed in a cumulative fashion to generate the experimental CDF. The CDF of a variable defines the probability of it being smaller than or equal to the value at which it is evaluated. In this context, the ESD CDF defines the empirical proportion of volume contained within any given distance to a selected structure. CDF of ESDs to different structures are represented as filled contours in which the central solid line depicts the average probability and the upper and lower bounds of the envelope represent the standard deviation. We studied the effect of image borders in the ESD by comparing its result when confined to different boundaries. We saw the effect was negligible, probably due to the dense occupation of all the cellular structures we study herein.

To study the spatial homogeneity of CARcs with respect to sinusoids we formulated the null hypothesis that the position of the detected cells *(x*_*i*_*)* was not influenced by their distance to the sinusoids (*Z(x*_*i*_*)*). Under this null hypothesis, *Z(x*_*i*_*)* should be a random sample of the ESD to the sinusoids (*Z(u)*). Two-sample Kolmogorov–Smirnov test was used to analyse significance.

### Data visualization

Imaris software was used to render confocal image stacks into 3D reconstructions and visualize the results generated through computational analysis in a virtual 3D space. A customized Imaris plugin (requiring ImarisXT module) was implemented in MATLAB to easily transfer images from Imaris to MATLAB for analysis. Processed and analysed images were then returned to Imaris for visualization as 3D rendering with OpenGL. 3D volumes were represented as a Maximum Intensity Projection with a perspective of 45°. Gamma correction was applied exclusively for visualization purposes. Binary images resulting from the segmentation were represented as a surface mesh in Imaris by making use of its surface segmentation utility. To represent the distribution of cells in the form of BM tissue maps we utilized the empirical probability density, which was calculated with a custom algorithm that adapted the kernel-based density estimation method to account for the different boundaries of the BM. The tissue map *f(u)* was calculated at every position *u* as defined in:

$$f\left( u \right) = \mathop {\sum}\limits_{i = 1}^n {K_\sigma \left( {u - x_i} \right)},$$

where *K*_*σ*_*(u)* is a 3D Gaussian kernel with standard deviation *σ* and a diameter of 8*σ* for each dimension. The value of *σ* was calculated for each image as the average nearest neighbour distance between the represented cells. The result was the 3D probability density function of the cell distribution. To generate 2D density maps as depicted in Fig. 2b and Supplementary Fig. 5d, the 3D map was averaged along the axial direction in the volume defined by the segmented DAPI mask.

### Statistical analysis of data

Data are expressed as mean ± SEM. Mann–Whitney tests were used to assess differences between groups unless stated otherwise. Kolmogorov–Smirnov test was employed to study the homogeneity of CARcs with respect to sinusoids in Figs. 6c and 7l. Statistical analysis was performed in MATLAB. No statistical method was used to predetermine sample size. Sample size was chosen based on prior studies and previously published literature.

### Code availability

The code for image analysis was implemented in Matlab as a set of user-friendly plugins for Imaris software (ImarisXT). Code and instructions for use can be found in Github https://github.com/alvgom/BM_3D-QM.

### Data availability

All raw data are available upon reasonable request to the corresponding author.

## Electronic supplementary material

Supplementary Information

Peer Review File

Description of Additional Supplementary Files

Supplementary Movie 1

Supplementary Movie 2

Supplementary Movie 3

Supplementary Movie 4

Supplementary Movie 5

Supplementary Movie 6

collagen IV CARc sinusoids

## References

[1] Nombela-Arrieta C, Manz MG (2017). Quantification and three-dimensional microanatomical organization of the bone marrow. Blood Adv..

[2] Kondo M (2003). Biology of hematopoietic stem cells and progenitors: implications for clinical application. Annu. Rev. Immunol..

[3] Takizawa H, Boettcher S, Manz MG (2012). Demand-adapted regulation of early hematopoiesis in infection and inflammation. Blood.

[4] Lee Y, Decker M, Lee H, Ding L (2017). Extrinsic regulation of hematopoietic stem cells in development, homeostasis and diseases. Wiley Interdiscip Rev. Dev. Biol..

[5] Morrison SJ, Scadden DT (2014). The bone marrow niche for haematopoietic stem cells. Nature.

[6] Mendelson A, Frenette PS (2014). Hematopoietic stem cell niche maintenance during homeostasis and regeneration. Nat. Med..

[7] Scadden DT (2012). Rethinking stroma: lessons from the blood. Cell Stem Cell.

[8] Calvi LM, Link DC (2014). Cellular complexity of the bone marrow hematopoietic stem cell niche. Calcif. Tissue Int..

[9] Mercier FE, Ragu C, Scadden DT (2012). The bone marrow at the crossroads of blood and immunity. Nat. Rev. Immunol..

[10] Crane GM, Jeffery E, Morrison SJ (2017). Adult haematopoietic stem cell niches. Nat. Rev. Immunol..

[11] Sivaraj KK, Adams RH (2016). Blood vessel formation and function in bone. Development.

[12] Hooper AT (2009). Engraftment and reconstitution of hematopoiesis is dependent on VEGFR2-mediated regeneration of sinusoidal endothelial cells. Cell Stem Cell.

[13] Butler JM (2010). Endothelial cells are essential for the self-renewal and repopulation of Notch-dependent hematopoietic stem cells. Cell Stem Cell.

[14] Rafii S, Butler JM, Ding BS (2016). Angiocrine functions of organ-specific endothelial cells. Nature.

[15] Kusumbe AP (2016). Age-dependent modulation of vascular niches for haematopoietic stem cells. Nature.

[16] Greenbaum A (2013). CXCL12 in early mesenchymal progenitors is required for haematopoietic stem-cell maintenance. Nature.

[17] Ding L, Saunders TL, Enikolopov G, Morrison SJ (2012). Endothelial and perivascular cells maintain haematopoietic stem cells. Nature.

[18] Ding L, Morrison SJ (2013). Haematopoietic stem cells and early lymphoid progenitors occupy distinct bone marrow niches. Nature.

[19] Kiel MJ (2005). SLAM family receptors distinguish hematopoietic stem and progenitor cells and reveal endothelial niches for stem cells. Cell.

[20] Nombela Arrieta C (2013). Quantitative imaging of haematopoietic stem and progenitor cell localization and hypoxic status in the bone marrow microenvironment. Nat. Cell Biol..

[21] Acar M (2015). Deep imaging of bone marrow shows non-dividing stem cells are mainly perisinusoidal. Nature.

[22] Kunisaki Y (2013). Arteriolar niches maintain haematopoietic stem cell quiescence. Nature.

[23] Itkin T (2016). Distinct bone marrow blood vessels differentially regulate haematopoiesis. Nature.

[24] Tokoyoda K, Egawa T, Sugiyama T, Choi BI, Nagasawa T (2004). Cellular niches controlling B lymphocyte behavior within bone marrow during development. Immunity.

[25] Pillai S, Cariappa A (2008). The bone marrow perisinusoidal niche for recirculating B cells and the positive selection of bone marrow-derived B lymphocytes. Immunol. Cell Biol..

[26] Junt T (2007). Dynamic visualization of thrombopoiesis within bone marrow. Science.

[27] Avecilla ST (2003). Chemokine-mediated interaction of hematopoietic progenitors with the bone marrow vascular niche is required for thrombopoiesis. Nat. Med..

[28] Pereira JP, An J, Xu Y, Huang Y, Cyster JG (2009). Cannabinoid receptor 2 mediates the retention of immature B cells in bone marrow sinusoids. Nat. Immunol..

[29] Kfoury Y, Scadden DT (2015). Mesenchymal cell contributions to the stem cell niche. Cell. Stem. Cell..

[30] Zhou BO, Yue R, Murphy MM, Peyer JG, Morrison SJ (2014). Leptin-receptor-expressing mesenchymal stromal cells represent the main source of bone formed by adult bone marrow. Cell Stem Cell.

[31] Yue R, Zhou BO, Shimada IS, Zhao Z, Morrison SJ (2016). Leptin receptor promotes adipogenesis and reduces osteogenesis by regulating mesenchymal stromal cells in adult bone marrow. Cell Stem Cell.

[32] Omatsu Y, Seike M, Sugiyama T, Kume T, Nagasawa T (2104). Foxc1 is a critical regulator of haematopoietic stem/ progenitor cell niche formation. Nature.

[33] Omatsu Y (2010). The essential functions of adipo-osteogenic progenitors as the hematopoietic stem and progenitor cell niche. Immunity.

[34] Sugiyama T, Kohara H, Noda M, Nagasawa T (2006). Maintenance of the hematopoietic stem cell pool by CXCL12-CXCR4 chemokine signaling in bone marrow stromal cell niches. Immunity.

[35] Asada N (2017). Differential cytokine contributions of perivascular haematopoietic stem cell niches. Nat. Cell Biol..

[36] Cordeiro Gomes A (2016). Hematopoietic stem cell niches produce lineage-instructive signals to control multipotent progenitor differentiation. Immunity.

[37] Kohara H (2007). Development of plasmacytoid dendritic cells in bone marrow stromal cell niches requires CXCL12-CXCR4 chemokine signaling. Blood.

[38] Noda M (2011). CXCL12-CXCR4 chemokine signaling is essential for NK-cell development in adult mice. Blood.

[39] Tokoyoda K, Hauser AE, Nakayama T, Radbruch A (2010). Organization of immunological memory by bone marrow stroma. Nat. Rev. Immunol..

[40] Sbalzarini, I. F. in *Focus on Bio-Image Informatics*, Advances in Anatomy, Embryology and Cell Biology Vol. 219 (eds De Vos, W. H., Munck, S. & Timmermans, J.-P.) 1–39 (Springer International Publishing, 2016).

[41] Helmuth JA, Paul G, Sbalzarini IF (2010). Beyond co-localization: inferring spatial interactions between sub-cellular structures from microscopy images. BMC Bioinforma..

[42] Domen J, Wagers AJ, Weissman IL (2006). Bone marrow (hematopoietic) stem cells. Regen. Med..

[43] Casanova-Acebes M (2013). Rhythmic modulation of the hematopoietic niche through neutrophil clearance. Cell.

[44] Smith-Berdan S, Nguyen A, Hong MA, Forsberg EC (2015). ROBO4-mediated vascular integrity regulates the directionality of hematopoietic stem cell trafficking. Stem Cell Rep..

[45] Kusumbe AP, Ramasamy SK, Adams RH (2014). Coupling of angiogenesis and osteogenesis by a specific vessel subtype in bone. Nature.

[46] Geiger H, de Haan G, Florian MC (2013). The ageing haematopoietic stem cell compartment. Nat. Rev. Immunol..

[47] Kovtonyuk LV, Fritsch K, Feng X, Manz MG, Takizawa H (2016). Inflamm-aging of hematopoiesis, hematopoietic stem cells, and the bone marrow microenvironment. Front. Immunol..

[48] Ambrosi TH (2017). Adipocyte accumulation in the bone marrow during obesity and aging impairs stem cell-based hematopoietic and bone regeneration. Cell Stem Cell.

[49] Stegner D (2017). Thrombopoiesis is spatially regulated by the bone marrow vasculature. Nat. Commun..

[50] Inra CN (2015). A perisinusoidal niche for extramedullary haematopoiesis in the spleen. Nature.

[51] Coutu DL, Kokkaliaris KD, Kunz L, Schroeder T (2017). Three-dimensional map of nonhematopoietic bone and bone-marrow cells and molecules. Nat. Publ. Group.

[52] Coutu DL, Kokkaliaris KD, Kunz L, Schroeder T (2017). Multicolor quantitative confocal imaging cytometry. Nat. Methods.

[53] Baddeley, A., Rubak, E. & Turner, R. *Spatial Point Patterns* (CRC Press, Boca Raton, 2015).

[54] Lagache T, Lang G, Sauvonnet N, Olivo-Marin JC (2013). Analysis of the spatial organization of molecules with robust statistics. PLoS One.

[55] Jammalamadaka A (2015). Characterizing spatial distributions of astrocytes in the mammalian retina. Bioinformatics.

[56] Ara T (2003). Long-term hematopoietic stem cells require stromal cell-derived factor-1 for colonizing bone marrow during ontogeny. Immunity.

[57] Boettcher S (2014). Endothelial cells translate pathogen signals into G-CSF-driven emergency granulopoiesis. Blood.

[58] Corada M (2013). Sox17 is indispensable for acquisition and maintenance of arterial identity. Nat. Commun..

[59] Bixel MG (2017). Flow dynamics and HSPC homing in bone marrow microvessels. Cell Rep..

[60] Sipkins DA (2005). In vivo imaging of specialized bone marrow endothelial microdomains for tumour engraftment. Nat. Publ. Group.

[61] Mazo IB (1998). Hematopoietic progenitor cell rolling in bone marrow microvessels: parallel contributions by endothelial selectins and vascular cell adhesion molecule 1. J. Exp. Med..

[62] Breitbach M (2018). In vivo labeling by CD73 marks multipotent stromal cells and highlights endothelial heterogeneity in the bone marrow niche. Cell Stem Cell.

[63] Bettelli E (2006). Reciprocal developmental pathways for the generation of pathogenic effector TH17 and regulatory T cells. Nature.

[64] Mueller SN, Germain RN (2009). Stromal cell contributions to the homeostasis and functionality of the immune system. Nat. Rev. Immunol..

[65] Chang JE, Turley SJ (2015). Stromal infrastructure of the lymph node and coordination of immunity. Trends Immunol..

[66] Choi JS, Harley BAC (2017). Marrow-inspired matrix cues rapidly affect early fate decisions of hematopoietic stem and progenitor cells. Sci. Adv..

[67] Nakamura-Ishizu A (2012). Extracellular matrix protein tenascin-C is required in the bone marrow microenvironment primed for hematopoietic regeneration. Blood.

[68] Steinert EM (2015). Quantifying memory CD8 T cells reveals regionalization of immunosurveillance. Cell.

[69] Schepers K (2013). Myeloproliferative neoplasia remodels the endosteal bone marrow niche into a self-reinforcing leukemic niche. Cell Stem Cell.

[70] Hanoun M (2014). Acute myelogenous leukemia-induced sympathetic neuropathy promotes malignancy in an altered hematopoietic stem cell niche. Cell Stem Cell.

[71] Terashima A (2016). Sepsis-induced osteoblast ablation causes immunodeficiency. Immunity.

[72] Bruns I (2014). Megakaryocytes regulate hematopoietic stem cell quiescence through CXCL4 secretion. Nat. Med..

[73] Mokhtari Z, Mech F, Zehentmeier S, Hauser AE, Figge MT (2015). Quantitative image analysis of cell colocalization in murine bone marrow. Cytometry.

[74] Shimoto M, Sugiyama T, Nagasawa T (2017). Numerous niches for hematopoietic stem cells remain empty during homeostasis. Blood.

