# Supplementary material for: Quantitative spatial analysis of haematopoiesis-regulating stromal cells in the bone marrow microenvironment by 3D microscopy
Source: Nat Commun. 2018 Jun 28;9:2532. doi: 10.1038/s41467-018-04770-z (PMC6023894; doi:10.1038/s41467-018-04770-z)
Supplement: Supplementary file 1 — Supplementary Information [file 41467_2018_4770_MOESM1_ESM.pdf]

Supplementary Information

Quantitative spatial analysis of hematopoiesis-regulating stromal cells in the bone marrow microenvironment by 3D microscopy

Gomariz, Helbling, Isringhausen et al.

Gomariz A., Helbling, P. Isringhausen,S. *et al.*

Supplemmentary Figure 1

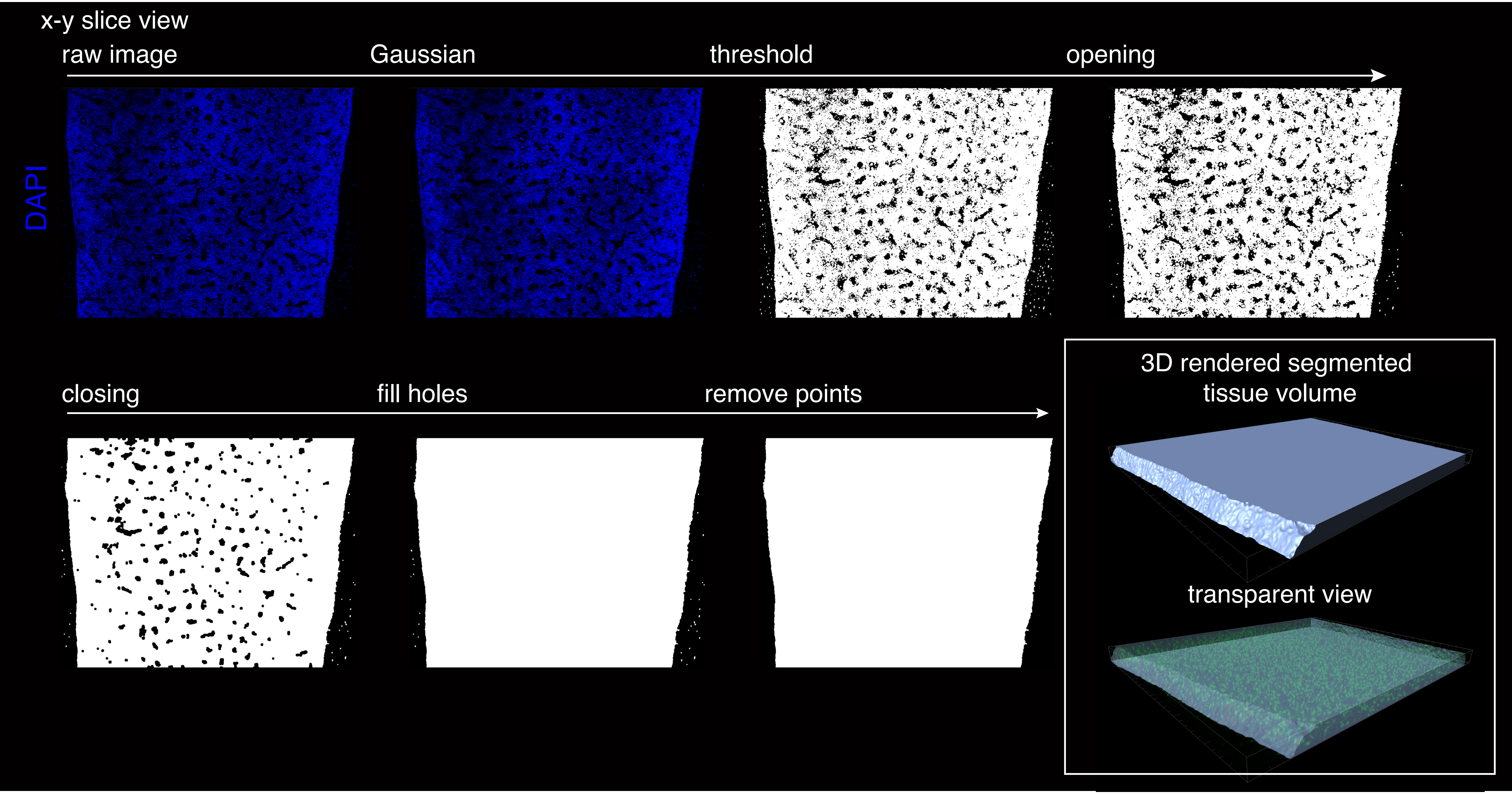

Supplementary Figure 1. Extraction of absolute tissue volumes for quantitative and spatial analysis of BM by 3D-QM. Sequence of image processing operations applied to the fluorescent DAPI signal to delimit and render the tissue regions in which objects are analyzed. The high-intensity DAPI nuclear signal, which homogeneously extends throughout the densely populated extravascular BM space, is employed to define the external borders of the tissue both in cortical and trabecular bone regions. Small areas with low DAPI signal corresponding to the wide intravascular volume of sinusoids are closed using a set morphological image processing operations serially performed in individual optical sections. In the pipeline shown here we applied Gaussian blurring with σ=5, a threshold to intensities higher than 15, opening with a circular kernel of radius 5 pixels, closing with a circular kernel of radius 15 pixels, region filling with an area threshold of 40000 pixels, and remove regions with an area threshold of 5000 pixels. Once processed, 2D binary image stacks are assembled to render 3D blocks of BM content. The computation of total tissue volume in regions of interest analyzed allows for estimation of global and regional densities of specific cell subsets. See also Supplementary Video 2 and Methods section.

Gomariz A., Helbling, P. Isringhausen,S. *et al.*

Supplemmentary Figure 2

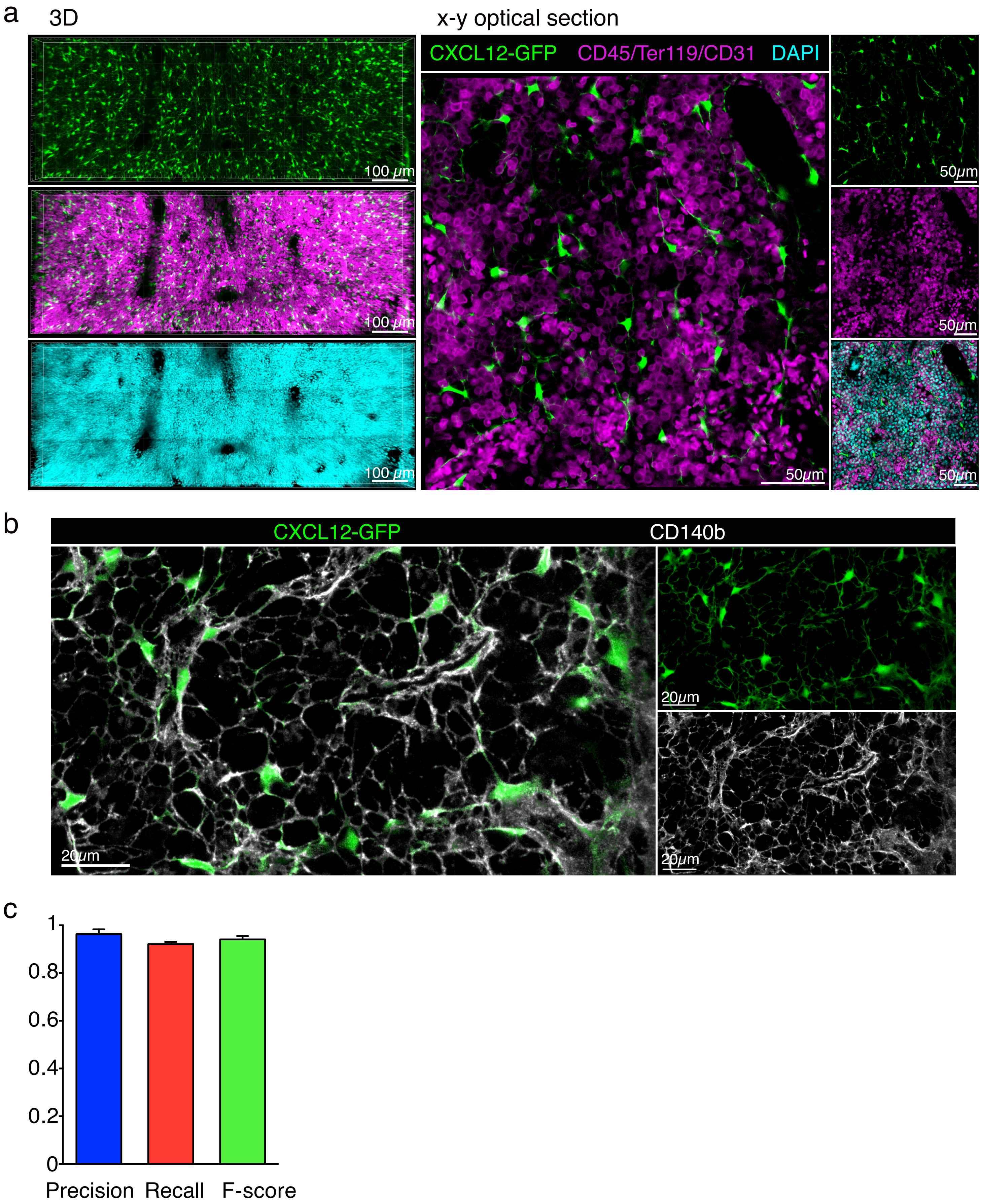

Supplementary Figure 2. Quantitative analysis of CARc in *Cxcl12-Gfp* mice. (a) CXCL12-GFP⁺ cells visualized and quantified in 3D imaging do not express hematopoietic or vascular markers. Left: low magnification image of femoral BM from *CXCL12-Gfp* mice immunostained for CD45/Ter119 (hematopoietic) and CD31 (vascular marker). Right: 2D slice of a zoomed-in region from image on the left. (b) Representative image showing co-expression of CD140b (white) in all GFP⁺ cells in BM of *Cxcl12-Gfp* mice. (c) Performance assessment of CARc automatic detection using the Imaris Spots module compared to manual annotation by three independent experienced researchers. Precision, recall, and F-score were calculated by comparing automatically detected cells with manually annotated ground truth. Evaluation of 567 detected cells resulted in a precision of 96.3±2.0%, a recall of 92.1±0.9%, and a F-score of 94.1±1.4%.Bars represent means +/- s.d.

Supplemmentary Figure 3

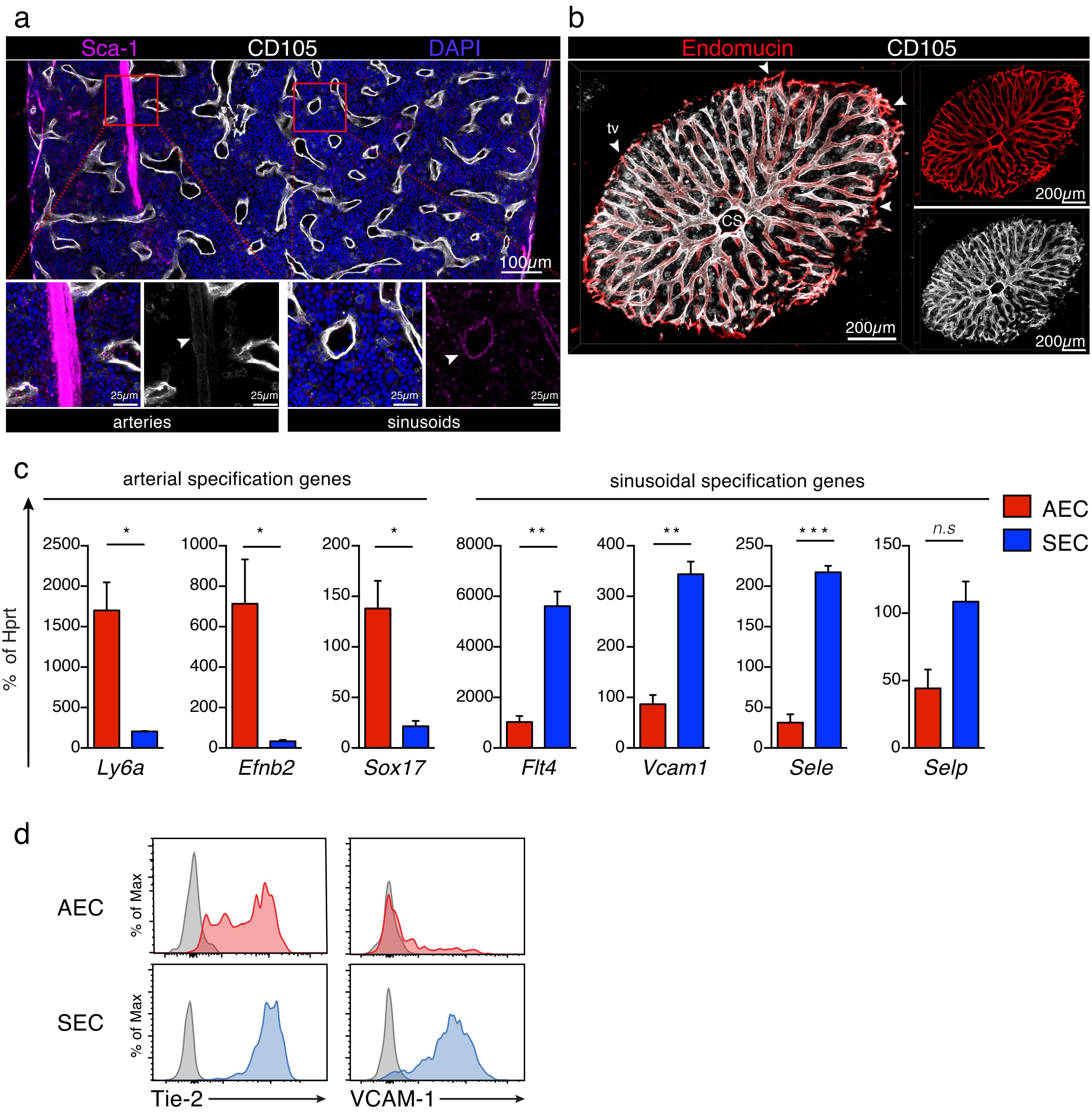

Supplementary Figure 3. Specific immunophenotypic profiles for BM arterial and sinusoidal endothelial cell identification by 3D-QM and FC. (a) Distinct expression of CD105 and Sca-1(Ly6a) in BM vascular types as observed by immunostaining of tissue sections. Zoomed-in images depict detailed views of arterial vessels expressing high levels of Sca-1 and moderate expression of CD105 (marked by arrowhead in bottom left panels). Sinusoidal vessels (bottom right) display strong CD105 and weak Sca-1 expression. **(b)** Immunostaining of CD105 and endomucin in a transversal 3D image at the level of the femoral diaphysis (cs: central sinus, tv: transitional vessel). Transitional vessels are found in the outer zone of the diaphyseal cavity, right along endosteal surfaces and connecting arterial and sinusoidal circulation. **(c)** Expression of prototypical genes involved in specification, identity or function of arterial and sinusoidal cells in flow sorted $CD45^-Ter119^-$ $CD31^+Sca\text{-}1^{hi}CD105^{int}$ arterial endothelial cells (AEC) and $CD45^-Ter119^-$ $CD31^+Sca\text{-}1^{int}CD105^{hi}$ sinusoidal endothelial cells (SEC) populations. Statistical significance was analyzed by Student's t test. * P< 0.05, ** P< 0.01, *** P< 0.005 **(d)** Differential expression levels of Tie-2 and VCAM-1 in AECs (red) and SECs (blue) as assessed by FC. Matched isotype control signals are shown in grey.

Supplemmentary Figure 4

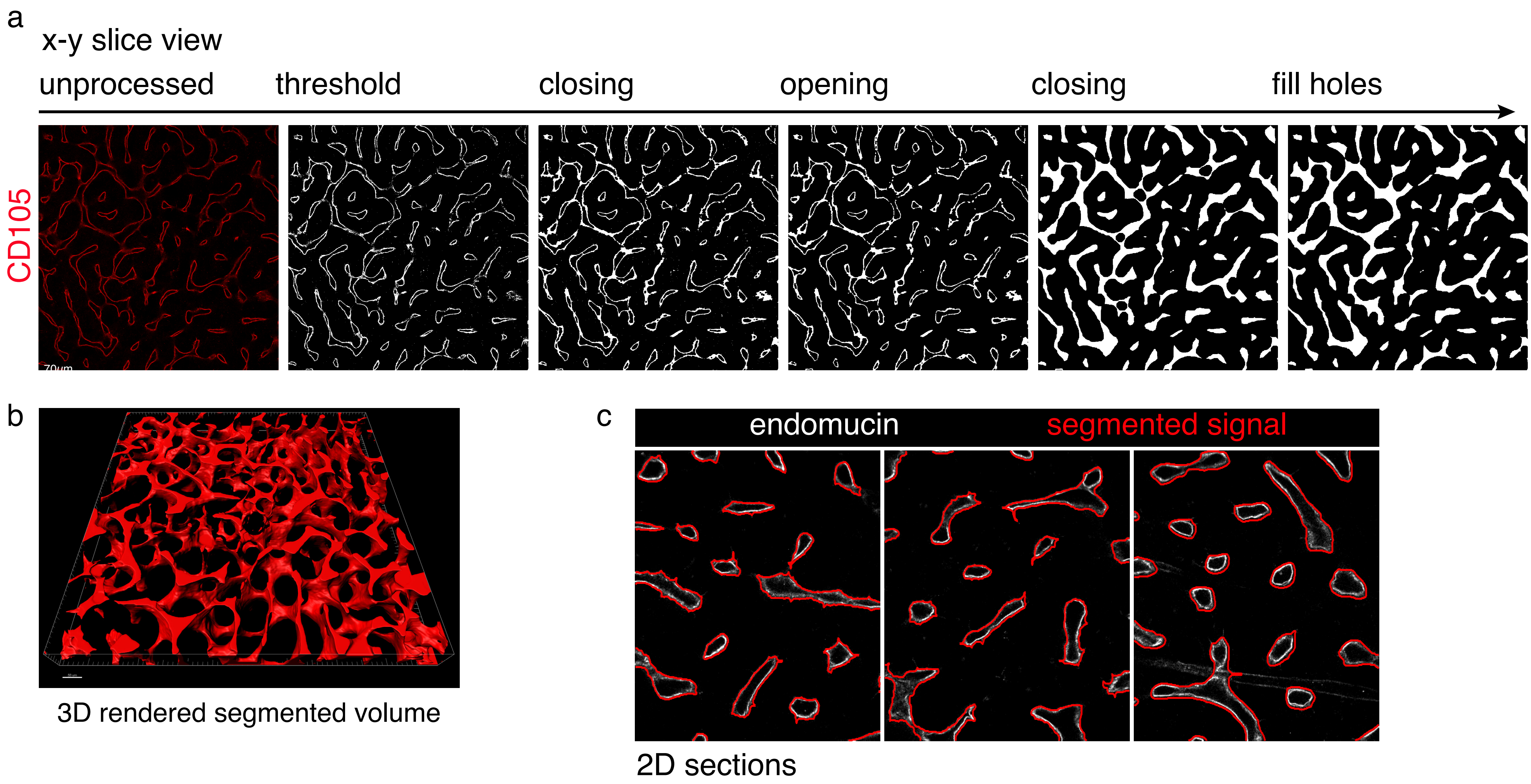

Supplementary Figure 4. 3D digital rendering of the sinusoidal vascular network by image processing.(a) Sequential application of morphological image processing operations in 2D optical sections permits detection of the fluorescent signal of sinusoidal vessel walls, and subsequent filling of the lumina of these wide vessels. Results after application of each operator are shown for one representative 2D optical section. In this image we used Otsu thresholding, a circular morphological image operator with different radii (3 μm for the first closing, 2 μm for opening, and 5 μm for the second closing), and region filling for enclosed areas smaller than 5000 pixels. The final operation consists of the assembly of all processed sections to obtain a 3D rendering of sinusoidal networks as solid tubular structures. **(b)** The final operation consists on the assembly of all processed sections to obtain a 3D rendering of sinusoidal networks as continuous tubular structures. **(c)** Qualitative evaluation of the segmentation method for representative regions in different samples. 2D slices are here included to provide a visual assessment of the performance of our methods for detection of sinusoids. The contour of the segmented sinusoids is displayed in red as an overlay of the white signal (grey scale) of endomucin. See also Methods section and Supplementary Movie 3.

Supplemmentary figure 5

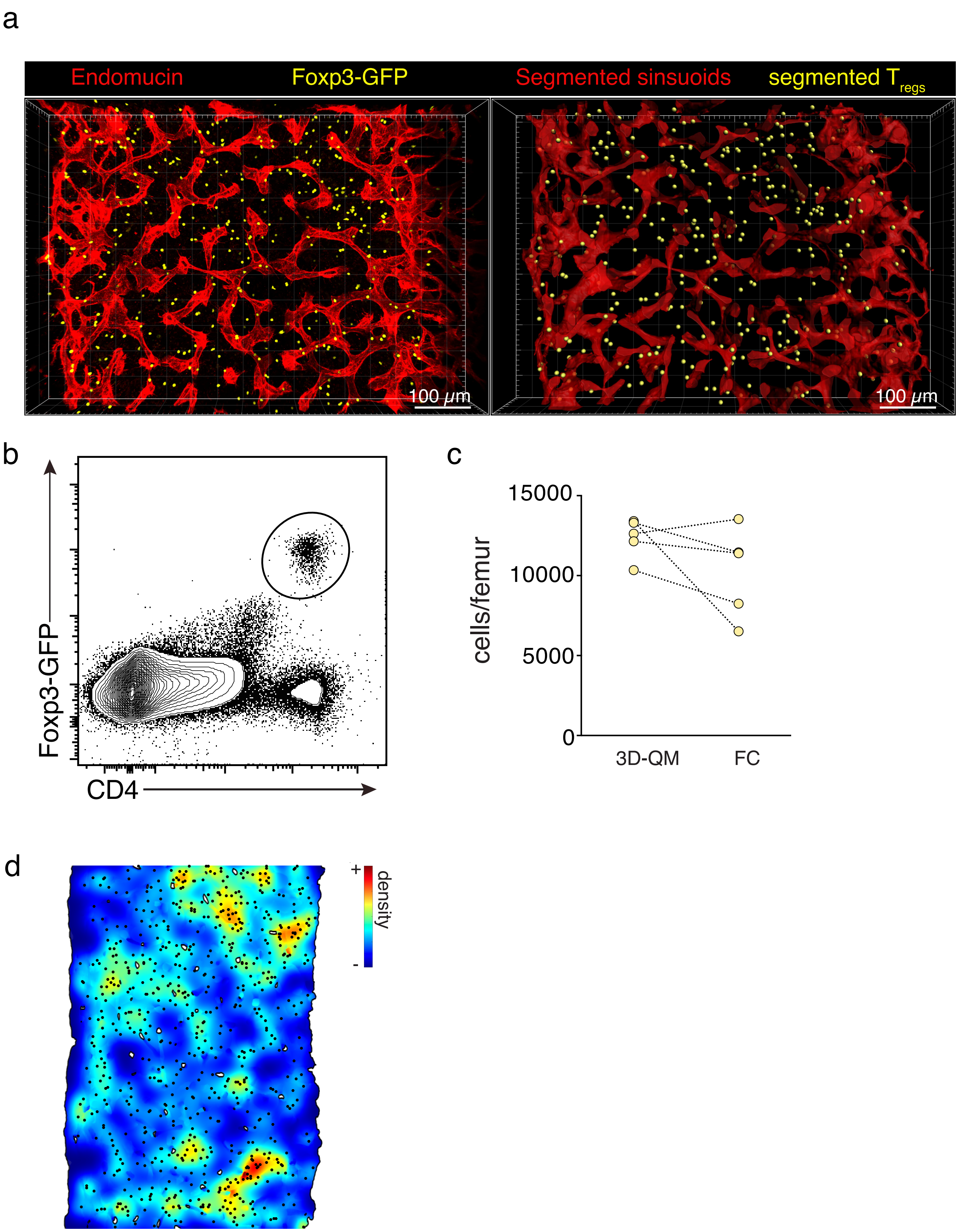

Supplementary Figure 5. Quantification of BM-resident regulatory T cells by 3D-QM and FC. (a) Visualization of GFP+ regulatory T cells (Tregs) in BM 3D images by confocal imaging of femoral cavities of Foxp3-Gfp mice. The left image depicts tissue reconstructions of unprocessed confocal image stacks. The imageon the right depicts segmented vascular structures and detected Tregs. **(b)** Gating strategy to detect Treg cells by FC as Foxp3-GFP+CD4+ cells. **(c)** Absolute numbers of Tregs quantified by FC and 3D quantitative microscopy in single femurs (n=5).**(d)** Tissue map depicting density and distribution of Tregs in one representative 3D image of a mouse femoral diaphysis.

Supplementary Table 1: List of reagents, commercial sources and catalog numbers if available.

**Antibodies flow cytometry**	**Commercial source**	**Cat.no**
Anti-Mouse CD45 PerCP-Cyanine5.5	eBioscience	45-0451-82
Anti-Mouse TER-119 PerCP-Cyanine5.5	eBioscience	45-5921-82
Anti-Mouse CD31 (PECAM-1) PE-Cyanine7	eBioscience	25-0311-82
Anti-Mouse CD140b (PDGF Receptor b) APC	eBioscience	17-1402-82
BV786 Rat Anti-Mouse CD105	BD Biosciences	564746
TruStain fcX™ (anti-mouse CD16/32)	BioLegend	101320
APC/Cy7 anti-mouse Ly-6A/E (Sca-1)	BioLegend	108126

**Antibodies immunohistology**	**Commercial source**	**Cat.no**
Endomucin	Santa Cruz Biotechnology	Sc-65495
CD105 / Endoglin	R&D Systems	AF1320
Laminin	Sigma	L9393
Perlecan	Thermo Fisher Scientific	MA5-14641
Collagen IV	Abcam	Ab6586
Fibronectin	Abcam	Ab2413
CD140b	eBioscience	13-1402-82
Ly-6A/E (Sca1)	BioLegend	122502
Alexa Fluor® 647 AffiniPure F(ab')$_2$ Fragment Donkey Anti-Rat IgG (H+L)	Jackson Immuno Research	712-606-153
Alexa Fluor® 594 AffiniPure Donkey Anti-Rabbit IgG (H+L)	Jackson Immuno Research	711-585-152
Cy™3 AffiniPure Donkey Anti-Goat IgG (H+L)	Jackson Immuno Research	705-165-147
Cy™3 AffiniPure F(ab')$_2$ Fragment Donkey Anti-Rabbit IgG (H+L)	Jackson Immuno Research	711-166-152
Alexa Fluor® 488 AffiniPure Donkey Anti-Goat IgG (H+L)	Jackson Immuno Research	705-545-147
Alexa Fluor 680 Donkey anti-Rabbit IgG (H+L) Highly Cross-Adsorbed Secondary Antibody	Thermo Fisher Scientific	A10043

**Chemicals/ machines / consumables**	**Commercial source**	**Cat.no**
RapiClear 1.52	SunJin Lab Co	RC152001
Collagenase, Type 2	Worthington Biochem.	LS004176
Deoxyribonuclease I	Worthington Biochem.	LS002007
PKH26 Reference Microbeads	Sigma-Aldrich	P7458-100ML
EDTA solution pH 8.0 (0.5 M) for molecular biology	Panreac AppliChem	A4892,0100
DMEM, high glucose, GlutaMAX™ supplement	Thermo Fisher Scientific	61965059
HEPES (1M)	Thermo Fisher Scientific	15630056
High-Capacity cDNA Reverse Transcription Kit	Thermo Fisher Scientific	4368814
7500 Fast Real-Time PCR System	Thermo Fisher Scientific	4351106
Power SYBR® Green PCR Master Mix	Thermo Fisher Scientific	4367659
TaqMan® Gene Expression Master Mix	Thermo Fisher Scientific	4369016
RNeasy Plus Micro Kit (50)	Qiagen	74034
Falcon™ Cell Strainers	Thermo Fisher Scientific	08-771-2
OCT	Leica Biosystems	14020108926
Parafomaldehyde	Electron Microscopy Sciences	15710
Trixon X	Sigma	X100-100ML
BSA	Sigma	A4503
Rapiclear	SunJin Lab	RC152001
Dow Corning® high-vacuum silicone grease	Sigma	Z273554-1EA

Supplementary Table 2: List of primer employed in RT-qPCR experiments including sequences and sources

**qPCR primers**		
Emcn	Thermo Fisher Scientific	Mm00497495_m1
Flt4	Thermo Fisher Scientific	Mm01292604_m1
Ephb2	Thermo Fisher Scientific	Mm01181021_m1
Hprt	Thermo Fisher Scientific	Mm03024075_m1
Selp for (TCCAGGAAGCTCTGACGTACTTG)	DOI: 10.1084/jem.20101545	
Selp rev (GCAGCGTTAGTGAAGACTCCGTAT)	DOI: 10.1084/jem.20101545	
Sele for (TGAACTGAAGGGATCAAGAAGACT)	DOI: 10.1084/jem.20101545	
Sele rev (GCCGAGGGACATCATCACAT)	DOI: 10.1084/jem.20101545	
Sox17 for (CACAACGCAGAGCTAAGCAA)	DOI: 10.1016/j.cell.2007.06.011	
Sox17 rev (CGCTTCTCTGCCAAGGTC)	DOI: 10.1016/j.cell.2007.06.011	
Eng for (GGTCATGACTCTGGCACTCA)	DOI: 10.1371/journal.pone.0086273	
Eng rev (AGGCGCTACTCAGGACAAGA)	DOI: 10.1371/journal.pone.0086273	
Ly6a for (TTTATCTGTGCAGCCCTTCTC)	-	
Ly6a rev (CAGTCCCTGAGCTCTTTCTG)	-	
Pecam1 for (TAGCAAGAAGCAGGAAGGAC)	-	
Pecam1 rev (GGCAAGGAAGACTCTGACTG)	-	
Hprt for (CTC TCG AAG TGT TGG ATA CAG)	-	
Hprt rev (ACA AAC GTG ATT CAA ATC CC)	-	

Supplementary Table 3. Objectives and image-acquisition settings for confocal microscopy

	**Lateral voxel size**	**Axial voxel size**
**10 x objective** (HC PL FLUOTAR, NA 0.30, WD 11.0)	1.5 μm	5 – 10 μm
**20 x objective** (HC PL APO CS2, NA 0.75, WD 0.68)	0.3 – 0.6 μm	0.75 – 1.5 μm
**63 x objective** (HC PL APO CS2, NA 1.4, WD 0.14)	0.19 μm	0.3 – 0.5 μm
**93 x objective** (HC PL APO motCORR, NA 1.3, WD 0.3)	0.12 μm	0.4 μm
