# Supplementary material for: Quantitative spatial analysis of haematopoiesis-regulating stromal cells in the bone marrow microenvironment by 3D microscopy
Source: Nat Commun. 2018 Jun 28;9:2532. doi: 10.1038/s41467-018-04770-z (PMC6023894; doi:10.1038/s41467-018-04770-z)
Supplement: Supplementary file 3 — Description of Additional Supplementary Files [file 41467_2018_4770_MOESM3_ESM.docx]

**Description of Additional Supplementary Files**

File Name: Supplementary Movie 1

Description**: Multiscale 3D-QM of BM tissues:** Confocal image stacks obtained from one single femoral bone from Cxcl12-Gfp mouse at different magnifications and resolutions ranging from organ-wide to cellular and subcellular scales. Registration of up to five fluorescent markers allow for simultaneous visualization of arterial and sinusoidal microvascular networks, mesenchymal cells, extracellular matrix fibres (highest resolution) and DAPI+ nuclei in the volumetric images displayed. Image-based computational analysis allows for segmentation and detection of objects for quantification.

File Name: Supplementary Movie 2

Description: **Multiscale 3D-QM of BM tissues:** Confocal image stacks obtained from one single femoral bone from Cxcl12-Gfp mouse at different magnifications and resolutions ranging from organ-wide to cellular and subcellular scales. Registration of up to five fluorescent markers allow for simultaneous visualization of arterial and sinusoidal microvascular networks, mesenchymal cells, extracellular matrix fibres (highest resolution) and DAPI+ nuclei in the volumetric images displayed. Image-based computational analysis allows for segmentation and detection of objects for quantification.

File Name: Supplementary Movie 3

Description: **3D digital rendering of the sinusoidal vascular network by image processing.** Image processing allows for the detection of endothelial outline (red, Endomucin-specific signal) of sinusoidal vessel walls. Lumina are filled through the application of morphological image processing operations for segmentation and reconstruction of vascular networks. For technical details see Supplementary Fig. 4 and Methods.

File Name: Supplementary Movie 4

Description: **Quantification of SEC numbers and cell densities in BM 3D images.** Z-stack scanning of x-y optical sections permits identification and annotation of SEC nuclei based on Endomucin (red) and DAPI (blue) staining. Individual SEC nuclei are labelled in in 2D images and visualized in 3D renders of BM microenvironment.

File Name: Supplementary Movie 5

Description: **Image-based quantification of regulatory T cell (Tregs) densities in BM.** Representative 3D image of BM from Foxp3-Gfp mice. Bright GFP expression allows for segmentation and quantification of Tregs (green) in large BM volumes. BM sinusoidal vessels are depicted in red.

File Name: Supplementary Movie 6

Description: **3D visualization of CAR-ECM network associations.** Representative 3D images of BM depicting extracellular matrix (ECM) fibres (white, immunostained for Collagen IV), sinusoidal vessels (immunostained for Endomucin and reconstructed as isosurfaces) and the CXCL12-GFP signal labelling CARc bodies, as well as their dense network of cytoplasmic projections emitted along ECM scaffolds.

File Name: Supplementary Movie 7

Description: **CARc significantly accumulate in direct contact with sinusoidal surfaces.** BM 3D image showing classification of two subpopulations of CARc (green) based on adjacency to sinusoidal surfaces (red). Spatial analysis demonstrates that the majority of CARc are found in direct contact with sinusoids (marked with green spheres towards the end of the video). A minor, but detectable fraction of cells is consistently observed as physically separated from sinusoidal surfaces (white spheres).
