# Supplementary material for: Quantitative spatial analysis of haematopoiesis-regulating stromal cells in the bone marrow microenvironment by 3D microscopy
Source: Nat Commun. 2018 Jun 28;9:2532. doi: 10.1038/s41467-018-04770-z (PMC6023894; doi:10.1038/s41467-018-04770-z)
Supplement: Supplementary file 2 — Peer Review File [file 41467_2018_4770_MOESM2_ESM.pdf]

Reviewers' comments:

Reviewer #1 (Remarks to the Author):

Gomariz and colleagues have used the clearing method and 3D microscopy to provide imaging of CXCL12-abundant reticular cells and the bone marrow vasculature. The quality of these imaging is great and the data suggesting that the numbers of CAR cells and sinusoidal endothelial cells are much higher than previously thought is of potential interest. In some ways, it is expected but this paper has clearly quantified it very well. The downside of the study is that most of the results are confirmatory of previous studies and descriptive. In the current state, and it is unclear whether their findings are relevant to the interplay of HSCs and their niche. Specific comments are as follows:

Major comments

1. It appears that different marker sets were used in experiments that compare the numbers of cells detected by 3D-QM and flow cytometric analyses. The same markers should be used in each comparison to correctly interpret the data.

2. Given that one of the major concerns in the stem cell biology is comparison of the numbers of stem cells and their niche, it will add some values to this study if the authors provide the information of the number of HSCs defined by their 3D-QM.

3. Recent studies have revealed that peri-arteriolar cells also play a role as HSC niche. Related to the comment above, it would be important to examine the number of peri-arteriolar cells with 3D-QM.

4. It would be interesting to investigate the interaction of HSCs and extracellular matrix in the bone marrow with the use of the authors' technique, which might lead to provide a novel mechanism for HSC regulation.

Minor comments

1. The term "3D-QM" should be defined.

Reviewer #3 (Remarks to the Author):

The paper is very well written, clear and exhaustive. It contain very useful elements for investigation of 3D spatial quantitative informations to be obtained also in other contexts, where spatial quantitative distribution analysis is required.

The analysis is also performed in a precise way and the figures obtained are impressive, together with the conclusions which highlight the importance of a 3D quantitative analysis.

Reviewer #4 (Remarks to the Author):

Gomariz et al. describe a very detailed, elegant, and highly quantitative analysis of some components of bone marrow stroma, and definitively demonstrate how flow cytometry is an inadequate technology to assess bone marrow stroma. Instead, they use confocal microscopy of

thick bone sections, and quantitative image analysis to produce definitive numbers quantifying the amount of endothelial and CAR cells and the relationship between the two cell types.
Overall the finding that there are more CAR and endothelial cells than flow cytometry would make one think is not really novel as it is well known that the bone marrow is very highly vascularised, and there are published images of sections from CAR reporter mice that show their thick network. However, this fact is too often overlooked and a whole publication dedicated to it is worthwhile.
I recommend addressing a few points to make the approach described truly useful to the community.

The authors characterize in detail endothelial cells, demonstrating that they are either sinusoidal or arteriolar, and could even distinguish transitional vessels, however they do very little to better characterize the CXCL-12 reporter. Immunohistological stainings of CXCL12 show patterns different from the thick network of GFP+ cells. The authors should include an immunostaining for CXCL12 and compare GFP expression with protein localization, as differences in the two patterns may have implications for haematopoiesis.

It also would be ideal if the authors could include some novel observation about bone marrow biology, for example comparing bones of control vs. one of stressed/ infected/ aged/ irradiated/ leukemic mice.

In the discussion, it should be pointed out that these findings are consistent with previous ones described by the Lin, Morrison and other groups, and relevant references included.

Other points:
The authors describe clearly how they process the tissues and analyse the images, however very little is said about the microscopy. What excitation lasers/emission filters were used, whether adjustments for depth were made etc.

Page 8. We reasoned corresponded to SECs and AECs, respectively – SECs and AECs were swapped. Are references presented for all markers used?

Please review the scale bars presented in the figures as they are not always consistent within a figure, or there may be the measure but not the bar.

Reviewer #1 (Remarks to the Author):

Gomariz and colleagues have used the clearing method and 3D microscopy to provide imaging of CXCL12-abundant reticular cells and the bone marrow vasculature. The quality of these imaging is great and the data suggesting that the numbers of CAR cells and sinusoidal endothelial cells are much higher than previously thought is of potential interest. In some ways, it is expected but this paper has clearly quantified it very well. The downside of the study is that most of the results are confirmatory of previous studies and descriptive. In the current state, and it is unclear whether their findings are relevant to the interplay of HSCs and their niche. Specific comments are as follows:

We thank the reviewer for highlighting the technical quality of our work. We do agree that our results partially confirm those presented in other studies. Nonetheless, we would like to emphasize the technical and conceptual novelty of the results presented here.

First, we outline a complex experimental and computational pipeline, which allows us to provide high-quality, detailed image-based cellular quantifications that have not been reported to date. Second, we further employ statistical models, which have not been previously used to analyse BM tissues, to understand stromal cell organization and spatial interactions in the marrow. Third, based on these quantifications, we draw biological insight on the mode of action of these cell types and their relevance on BM physiology. Fourth, we have now included additional novel data on aged bone marrow. Finally our study reveals and calls for attention to a very important issue for the research field, namely that endothelial and mesenchymal populations that have been isolated and studied to date *ex vivo*, in fact represent only a really minor fraction of the native *in vivo* BM microenvironment.

Major comments

1. It appears that different marker sets were used in experiments that compare the numbers of cells detected by 3D-QM and flow cytometric analyses. The same markers should be used in each comparison to correctly interpret the data.

Response: We agree on the importance of the point raised by the reviewer. While we understand the use of different sets of markers can in principle be of concern, we do consider that we have provided compelling evidence to demonstrate that the strategies employed for subset identification with flow cytomery (FC) and 3D-QM are in fact strictly comparable, and therefore the populations analysed are equivalent. The reason for the minor divergence in sets of markers lies in the differences in sensitivity of detection, as well as in the reagents and type of staining methods required for each technology. A detailed explanation for both subpopulations analysed is provided below.

First, specific identification of **CARc** by FC was achieved by gating CD45$^-$Ter119$^-$CD31$^-$CD140b$^+$GFP$^+$ cells in CXCL12GFP mice. The inclusion of hematopoietic (CD45/Ter119) and endothelial (CD31) antigens as negative markers in the phenotypic signature was required to allow exclusion cell subsets of these origins, which express low levels of the CXCL12-GFP transgene

(Quintana et al., 2015) and data not shown) and may therefore “contaminate” the CARc gate. As shown in Figure 2g, once hematopoietic and endothelial cells are gated out, the remaining GFP^{+} cells express the mesenchymal marker CD140b. Therefore we are positive that these cells correspond to bona-fide CARc. Due to attenuated signal intensities in 3D specimens and higher detection thresholds typical of microscopy methods compared to FC, only cells expressing highest levels of CXCL12-GFP are directly visible in 3D-QM. Indeed, the lower intensity of the GFP signal in endothelial or hematopoietic cells was not observed, and therefore the use of specific markers to negatively gate out these cells could be avoided. In fact, in the initial version of the paper we showed that all of the detected GFP^{+} cells in 3D-QM expressed CD140b (just like in FC) and displayed the prototypical fibroblastic reticular morphology of mesenchymal networks (Supplementary Figure 2b). We have now included additional data, which clearly shows that GFP^{+} cells quantified by imaging, do not express CD45, Ter119, or CD31 cells (Supplementary Figure 2a of revised version). Collectively, the data demonstrates that CARc visualized in images are effectively CD45^{-}Ter119^{-}CD31^{-}CD140b^{+}GFP^{+}, and largely correspond to the same population as the one detected in FC analyses.

Second, for the quantification by FC of **sinusoidal endothelial cells (SEC)**, we employed the phenotypic signature CD45^{-}Ter119^{-}CD31^{+}CD105^{hi}Sca-1^{int}. Here we also stained for CD45 and Ter119 to exclude all hematopoietic, including erythroid cells, from our analysis. In turn, CD31 was employed to label all endothelial cells within the non-hematopoietic CD45^{-}Ter119^{-} gate. In BM images, comparable identification of endothelial cells can be directly achieved through immunostaining with one single highly specific endothelial cell marker (shown in Figure 3E). Indeed, the distinct morphological features of immunostained endothelial cells and their integration in the linings of continuous vascular networks, allow to unequivocally detect these endothelial cell types and easily distinguish them from round and small hematopoietic cells without the use of CD45 and Ter119 as negative markers. As a specific vascular antigen for imaging we chose the well-established marker endomucin over CD31. We did so for two reasons. a) staining for CD31 generated a very faint signal in sinusoidal endothelium using our staining protocols. b) CD31 is expressed by multiple hematopoietic cell types in the BM including megakaryocytes, platelets, and lymphocytes, some of which reside in close contact with sinusoids. Thus, the fluorescent signal of perisinusoidal cells would physically overlap with that of adjacent endothelial cells making it virtually impossible to precisely segment boundaries of endothelial linings. In contrast, immunostaining for endomucin provided a highly specific, high-intensity and distinct signal in vascular structures, amenable to our computational analysis (we refer to Figure 3 and Supplementary Figure 3) (Kwak et al., 2016). Once endothelial linings were identified in images we further employed the exact same Sca-1^{int}CD105^{hi} signature to discern the endothelial lining of sinusoidal walls and quantify the nuclei of SECs (which were CD105^{hi}Sca-1^{lo} and endomucin^{+}) (Supplementary Figure 3a and b).
Collectively we are confident that our strategies for identification of SECs and CARc are highly consistent between both methodologies. We hope this addresses the concerns of the reviewer and that the changes introduced enhance the clarity of the manuscript in this specific point.

2. Given that one of the major concerns in the stem cell biology is comparison of the numbers of

stem cells and their niche, it will add some values to this study if the authors provide the information of the number of HSCs defined by their 3D-QM.

Response: We agree that the anatomical localization of HSCs is a topic of great interest, and in fact remains one major unresolved question in hematopoiesis-related research. Conflicting results have been reported by different groups, mostly due to the significant technical challenges associated to simultaneously and reliably visualizing sufficient numbers of HSCs and interacting cell types in an intact tissue context. Among them the most prominent is most likely the fact that the complex combinations of surface markers required for identification of HSCs in FC are not easily amenable to histological sections and even less in the case of thick 3D specimens. For this reason a variety of mouse models in which individual HSCs can be identified through expression of one single fluorescent reporter have been recently generated (Acar et al., 2015; Chen et al., 2016; Gazit et al., 2014; Sawai et al., 2016). While some of these mouse strains are very useful, in our opinion only one laboratory has convincingly established methods for 3D imaging in the BM and employed them to comprehensively analyze HSC niches in a well-characterized reporter mouse strain (Acar et al., 2015). Given the high quality of our imaging strategy we definitely would like to address this type of investigation in the future, for which we are currently in the process of gaining access to some of the mouse strains generated in recent times. Considering the relevance of the question, the existing controversies, as well as the reagents, workload and time required to deliver a nuanced analysis of HSC niches, such type of in depth study would constitute a whole independent manuscript in itself. We would like to point out that our study is not intended to focus on HSC niches but rather addresses what, in our view, are very relevant questions on BM stromal cell infrastructure and organization.

3. Recent studies have revealed that peri-arteriolar cells also play a role as HSC niche. Related to the comment above, it would be important to examine the number of peri-arteriolar cells with 3D-QM.

Response: We thank the reviewer for this suggestion. Following our line of argumentation above, we consider that the specific interactions of HSCs with periarteriolar cells fall out of the topic of our study. Nevertheless, as pointed out by the reviewer, periarterial and periarteriolar microenvironments in the BM are known to harbour a distinct population of stromal cells of mesenchymal origin (periarteriolar MSCs) and have been recently proposed to constitute functionally distinct niches, not only for HSC maintenance but also for the distinct regulation of megakaryocytes (Itkin et al., 2016; Kunisaki et al., 2013; Tamura et al., 2016). Thus, in the context of our study we find it relevant to determine the size and distribution of periarterial niches. Accordingly, we have now additionally analysed the spatial arrangement of arterial and arteriolar networks with respect to the extravascular volume of BM tissues. For these experiments we employed the arterial-specific Sca-1 signal (Hooper et al., 2009; Itkin et al., 2016; Nombela Arrieta et al., 2013) to segment arteries and arterioles instead of periarteriolar stromal populations, for which no unique specific marker set has been reported. To date, labelling of certain periarteriolar

stromal subpopulations has been described in a variety of transgenic mouse models, including Nestin-GFP (as the NestinGFP^{hi} population) and in NG2Cre-ERT2 and Myh11-CreERT2 mice (Asada et al., 2017; Kunisaki et al., 2013). Nonetheless, these reporter lines do not provide the means to unambiguously identify periarteriolar populations. Furthermore, it is also unclear to what extent the subsets labelled in the different mouse strains in fact overlap. Given that by definition these cell types lie tightly adhered to arterial/arteriolar structures, we are confident that the arterial signal allows us to spatially define these niches in a very strict manner. We have included the new data in Figure 5g and 5h of the revised version of the manuscript. As expected, our results indicate that periarterial niches are much less frequent and more heterogeneously distributed than sinusoidal vessels, with only a minor fraction of the BM space (5.6%) being adjacent to these structures. As a consequence, access to and interactions with these microdomains is much more restricted than in the case of sinusoids and CARc.

4. It would be interesting to investigate the interaction of HSCs and extracellular matrix in the bone marrow with the use of the authors' technique, which might lead to provide a novel mechanism for HSC regulation.

Response: We are in full agreement that the essential roles of the ECM in the control of HSC biology and BM hematopoietic function remain largely underexplored to date. One of the main reasons for this likely lies in the fact that the detailed visualization of individual ECM fibers at sufficient resolution and in 3D had not been achieved until now. Our data supports critical and pleiotropic roles of ECM as it strongly suggests that all HSCs are in direct contact with this ubiquitous ECM network. However, whether ECM composition varies between different microdomains of the BM remains currently unknown and will certainly be instrumental to dissect out the diverse and specific roles of this network in hematopoiesis. As argued above, we believe that the functional analysis of HSC-ECM relationships clearly goes beyond the scope of the work reported here, and will for sure be addressed in the future with 3D techniques, as well as through the generation of novel mouse models deficient in ECM proteins.

Minor comments

1. The term "3D-QM" should be defined.

Response: We thank the reviewer for the correction and we have now defined the term 3D-QM, as "three-dimensional quantitative microscopy" the first time that it is stated in the manuscript.

Reviewer #3 (Remarks to the Author):

The paper is very well written, clear and exhaustive. It contain very useful elements for investigation of 3D spatial quantitative informations to be obtained also in other contexts, where spatial quantitative distribution analysis is required. The analysis is also performed in a precise way and the figures obtained are impressive, together with the conclusions, which highlight the importance of a 3D quantitative analysis.

We highly appreciate the positive comments from the reviewer. In the revised manuscript we provide additional data, which we trust the reviewer will find of relevance.

Reviewer #4 (Remarks to the Author):

Gomariz et al. describe a very detailed, elegant, and highly quantitative analysis of some components of bone marrow stroma, and definitively demonstrate how flow cytometry is an inadequate technology to assess bone marrow stroma. Instead, they use confocal microscopy of thick bone sections, and quantitative image analysis to produce definitive numbers quantifying the amount of endothelial and CAR cells and the relationship between the two cell types. Overall the finding that there are more CAR and endothelial cells than flow cytometry would make one think is not really novel as it is well known that the bone marrow is very highly vascularised, and there are published images of sections from CAR reporter mice that show their thick network. However, this fact is too often overlooked and a whole publication dedicated to it is worthwhile.I recommend addressing a few points to make the approach described truly useful to the community.

We are thankful to the reviewer for the positive comments and for highlighting the significance of our findings. We have included additional data to substantiate novelty.

The authors characterize in detail endothelial cells, demonstrating that they are either sinusoidal or arteriolar, and could even distinguish transitional vessels, however they do very little to better characterize the CXCL-12 reporter. Immunohistological stainings of CXCL12 show patterns different from the thick network of GFP+ cells. The authors should include an immunostaining for CXCL12 and compare GFP expression with protein localization, as differences in the two patterns may have implications for haematopoiesis.

Response: We agree on the importance of understanding the spatial distribution of CXCL12 within intact BM. Accessibility to this chemokine in BM tissues most likely influences migration, adhesion and cellular functions, critical for the progression along multiple stages of hematopoietic development. Nonetheless, we would like to emphasize that for the purpose of our study we solely employ GFP expression as a means to visualize, quantify and analyze spatial features of a certain specific cell type (CARc), which is labelled in the CXCL12GFP mice. We do not draw any conclusions on the relative contribution of this cell type as a source of CXCL12 nor of any other hematopoietic factor. Extensive previous work by multiple laboratories has substantiated the critical roles of CARc in hematopoietic regulation and HSC maintenance through production different factors, including CXCL12 but also SCF and IL-7 (Cordeiro Gomes et al., 2016; Ding and Morrison, 2013; Ding et al., 2012; Greenbaum et al., 2013). Given this proven relevance of CARc, our work focuses exclusively on the spatial interactions and abundance of this cell type. Thus, to what extent the expression reporter overlaps with the presence of CXCL12 protein, or what is the specific distribution of CXCL12 (whether secreted or intracellular) in BM, despite being a fundamental question for BM physiology, is in our view not critical for our study, nor does it affect the conclusions we draw.

To still address the reviewer's request, we have attempted to detect CXCL12 protein in the BM

using 3D-microscopy. Unfortunately, we were thus far not successful in our approach. Also, we are unaware of any publication in which such CXCL12 staining has been convincingly achieved in histological sections, given that the soluble or ECM-bound fractions of the protein are most likely lost during tissue processing and fixation.

Paul Frenette's group recently demonstrated through intracellular staining and FC analysis, that multiple cells, other than CARc, express significant levels of CXCL12 protein (Asada et al., 2017). Considering that different cellular sources of CXCL12 exist in the BM, and that the concentration of this chemokine in different tissue regions will not only depend on its production but also on its secretion, the mechanisms for its presentation in cells and ECM, and the degradation of the protein, the pattern of CXCL12 distribution in BM is most likely highly complex. As pointed out by the reviewer this remains an elusive and technically challenging question to address. Importantly, we would like to clarify that in our manuscript we do not imply that CARc are the sole producers of CXCL12.

It also would be ideal if the authors could include some novel observation about bone marrow biology, for example comparing bones of control vs. one of stressed/ infected/ aged/ irradiated/ leukemic mice.

Response: We agree with the reviewer's comment that inclusion of quantitative data on the BM under stress conditions would increase the interest and enhance the biological relevance of the paper. Following this suggestion we now provide in the revised manuscript a comprehensive characterization of CARc and sinusoidal networks in the bone marrow of aged mice (20-24 months old) using 3D-QM. The new data, which are presented in Figure 7 of the revised version of the manuscript, demonstrate that the maintenance of the structural integrity of the stromal components analysed is rather robust during homeostatic ageing. Notably, the densities of CARc, the volume of sinusoids and key parameters of CARc-sinusoidal interactions are largely comparable between young and aged mice. These findings are to some extent unexpected and therefore highlight the presence of solid mechanisms for structural maintenance in the BM. We modified the introduction, results and discussion accordingly and comment on the possible implications for ageing and hematopoiesis.

In the discussion, it should be pointed out that these findings are consistent with previous ones described by the Lin, Morrison and other groups, and relevant references included.

Response: We thank the reviewer for correctly pointing this out. It was our intention to appropriately credit previous results by other groups, which were cited in the initial version of the manuscript. More specifically we had highlighted the consistency of our work with that of the Morrison lab using 3D imaging of HSCs (Acar et al., 2015). Even though different methods were employed for sample preparation, computational image-based quantification and statistical analyses, when analysed in detail, the results obtained in the referred study, are well in line with our own findings in what refers to spatial interactions of CARc and sinusoids. We have modified the text of the discussion to emphasize the consistency of our study with that of Morrison and colleagues. In addition,

throughout the manuscript and more specifically in the discussion, we mention and comment on recent relevant work from multiple groups on the use of imaging techniques to explore cellular content and interactions in the BM (Acar et al., 2015; Bruns et al., 2014; Cordeiro Gomes et al., 2016; Coutu et al., 2017; Kunisaki et al., 2013; Mokhtari et al., 2015; Shimoto et al., 2017).

Other points: The authors describe clearly how they process the tissues and analyse the images, however very little is said about the microscopy. What excitation lasers/emission filters were used, whether adjustments for depth were made etc.

Response: We revised the Methods section accordingly and now provide a detailed description of the imaging procedures as per the reviewer's request (see subsection on Confocal Imaging). The settings for image acquisition using different objectives are also detailed in a new table in the revised version (Supplementary Table 3).

Page 8. We reasoned corresponded to SECs and AECs, respectively – SECs and AECs were swapped. Are references presented for all markers used?

Response: We thank the reviewer for pointing this out and apologize for this mistake. The terms SECs and AECs were not written in the correct order in the sentence indicated. We have now amended the text accordingly. In the revised version, we have also included new references for the markers used to identify both endothelial subsets. Specifically, in a recent manuscript Breitbach and colleagues show that E- selectin, P-Selectin and VCAM-1 are expressed at high levels in SECs than non-sinusoidal endothelial cells (Breitbach et al., 2018). This work supports classical studies, which demonstrated that homing of hematopoietic progenitor cells to the BM takes place in sinusoidal vessels expressing vascular selectins and VCAM-1 (Mazo et al., 1998; Sipkins et al., 2005). We included these references in the revised manuscript.

Please review the scale bars presented in the figures as they are not always consistent within a figure, or there may be the measure but not the bar.

Response: We thank the reviewer for pointing this out and have now revised all scale bars in the images presented to make sure that in all cases we depict the correct size and measure of scale bars.

References mentioned in the point by point response:

Acar, M., Kocherlakota, K.S., Murphy, M.M., Peyer, J.G., Oguro, H., Inra, C.N., Jaiyeola, C., Zhao, Z., Luby-Phelps, K., Morrison, S.J., 2015. Deep imaging of bone marrow shows non-dividing stem cells are mainly perisinusoidal. Nature 526, 126–130. doi:10.1038/nature15250
Asada, N., Kunisaki, Y., Pierce, H., Wang, Z., Fernandez, N.F., Birbrair, A., Ma'ayan, A., Frenette, P.S., 2017. Differential cytokine contributions of perivascular haematopoietic stem cell niches. Nat. Cell Biol. 1–19. doi:10.1038/ncb3475
Breitbach, M., Kimura, K., Luis, T.C., Fuegemann, C.J., Woll, P.S., Hesse, M., Facchini, R., Rieck, S., Jobin, K., Reinhardt, J., Ohneda, O., Wenzel, D., Geisen, C., Kurts, C., Kastenmüller, W.,

Hölzel, M., Jacobsen, S.E.W., Fleischmann, B.K., 2018. In Vivo Labeling by CD73 Marks Multipotent Stromal Cells and Highlights Endothelial Heterogeneity in the Bone Marrow Niche. Cell Stem Cell 22, 262–276.e7. doi:10.1016/j.stem.2018.01.008
Bruns, I., Lucas, D., Pinho, S., Ahmed, J., Lambert, M.P., Kunisaki, Y., Scheiermann, C., Schiff, L., Poncz, M., Bergman, A., Frenette, P.S., 2014. Megakaryocytes regulate hematopoietic stem cell quiescence through CXCL4 secretion. Nature Medicine 20, 1315–1320. doi:10.1038/nm.3707
Chen, J.Y., Wang, S.K., Yamazaki, S., Sinha, R., Kao, K.S., Seita, J., Sahoo, D., Nakauchi, H., Miyanishi, M., Weissman, I.L., 2016. Hoxb5 marks long-term haematopoietic stem cells and reveals a homogenous perivascular niche. Nature 530, 223–227. doi:10.1038/nature16943
Cordeiro Gomes, A., Hara, T., Lim, V.Y., Herndler-Brandstetter, D., Nevius, E., Sugiyama, T., Tani-ichi, S., Schlenner, S., Richie, E., Rodewald, H.-R., Flavell, R.A., Nagasawa, T., Ikuta, K., Pereira, J.P., 2016. Hematopoietic Stem Cell Niches Produce Lineage-Instructive Signals to Control Multipotent Progenitor Differentiation. Immunity 45, 1219–1231. doi:10.1016/j.immuni.2016.11.004
Coutu, D.L., Kokkaliaris, K.D., Kunz, L., Schroeder, T., 2017. Multicolor quantitative confocal imaging cytometry. Nat Meth 15, 39–46. doi:10.1038/nmeth.4503
Ding, L., Morrison, S.J., 2013. Haematopoietic stem cells and early lymphoid progenitors occupy distinct bone marrow niches. Nature 495, 231–235. doi:10.1038/nature11885
Ding, L., Saunders, T.L., Enikolopov, G., Morrison, S.J., 2012. Endothelial and perivascular cells maintain haematopoietic stem cells. Nature 481, 457–462. doi:10.1038/nature10783
Gazit, R., Mandal, P.K., Ebina, W., Ben-Zvi, A., Nombela-Arrieta, C., Silberstein, L.E., Rossi, D.J., 2014. Fgd5 identifies hematopoietic stem cells in the murine bone marrow. Journal of Experimental Medicine 107, 2317. doi:10.1016/j.stem.2011.07.003
Greenbaum, A., Hsu, Y.-M.S., Day, R.B., Schuettpelz, L.G., Christopher, M.J., Borgerding, J.N., Nagasawa, T., Link, D.C., 2013. CXCL12 in early mesenchymal progenitors is required for haematopoietic stem-cell maintenance. Nature 495, 227–230. doi:10.1038/nature11926
Hooper, A.T., Butler, J.M., Nolan, D.J., Kranz, A., Iida, K., Kobayashi, M., Kopp, H.-G., Shido, K., Petit, I., Yanger, K., James, D., Witte, L., Zhu, Z., Wu, Y., Pytowski, B., Rosenwaks, Z., Mittal, V., Sato, T.N., Rafii, S., 2009. Engraftment and reconstitution of hematopoiesis is dependent on VEGFR2-mediated regeneration of sinusoidal endothelial cells. Cell Stem Cell 4, 263–274. doi:10.1016/j.stem.2009.01.006
Itkin, T., Gur-Cohen, S., Spencer, J.A., Schajnovitz, A., Ramasamy, S.K., Kusumbe, A.P., Ledergor, G., Jung, Y., Milo, I., Poulos, M.G., Kalinkovich, A., Ludin, A., Kollet, O., Shakhar, G., Butler, J.M., Rafii, S., Adams, R.H., Scadden, D.T., Lin, C.P., Lapidot, T., 2016. Distinct bone marrow blood vessels differentially regulate haematopoiesis. Nature 532, 323–328. doi:10.1038/nature17624
Kunisaki, Y., Bruns, I., Scheiermann, C., Ahmed, J., Pinho, S., Zhang, D., Mizoguchi, T., Wei, Q., Lucas, D., Ito, K., Mar, J.C., Bergman, A., Frenette, P.S., 2013. Arteriolar niches maintain haematopoietic stem cell quiescence. Nature 502, 637–643. doi:10.1038/nature12612
Kwak, H., Salvucci, O., Weigert, R., Martinez-Torrecuadrada, J.L., Henkemeyer, M., Poulos, M.G., Butler, J.M., Tosato, G., 2016. Sinusoidal ephrin receptor EPHB4 controls hematopoietic progenitor cell mobilization from bone marrow. Journal of Clinical Investigation 1–15. doi:10.1172/JCI87848
Mazo, I.B., Gutierrez-Ramos, J.C., FRENETTE, P.S., Hynes, R.O., Wagner, D.D., Andrian, von, U.H., 1998. Hematopoietic progenitor cell rolling in bone marrow microvessels: parallel contributions by endothelial selectins and vascular cell adhesion molecule 1. J. Exp. Med. 188, 465–474.
Mokhtari, Z., Mech, F., Zehentmeier, S., Hauser, A.E., Figge, M.T., 2015. Quantitative image analysis of cell colocalization in murine bone marrow. Cytometry 87, 503–512. doi:10.1002/cyto.a.22641
Nombela Arrieta, C., Pivarnik, G., Winkel, B., Canty, K.J., Harley, B., Mahoney, J.E., Park, S.-Y.,

Lu, J., Protopopov, A., Silberstein, L.E., 2013. Quantitative imaging of haematopoietic stem and progenitor cell localization and hypoxic status in the bone marrow microenvironment. Nat. Cell Biol. 15, 533–543. doi:10.1038/ncb2730

Quintana, J.A., Ligos, J.E.M., Leiva, M., Hidalgo, A.E.S., 2015. Haematopoietic ESL-1 enables stem cell proliferation in the bone marrow by limiting TGF. Nature Communications 7, 1–10. doi:10.1038/ncomms10222

Sawai, C.M., Babovic, S., Upadhaya, S., Knapp, D.J.H.F., Lavin, Y., Lau, C.M., Goloborodko, A., Feng, J., Fujisaki, J., Ding, L., Mirny, L.A., Merad, M., Eaves, C.J., Reizis, B., 2016. Hematopoietic Stem Cells Are the Major Source of Multilineage Hematopoiesis in Adult Animals. Immunity 1–26. doi:10.1016/j.immuni.2016.08.007

Shimoto, M., Sugiyama, T., Nagasawa, T., 2017. Numerous niches for hematopoietic stem cells remain empty during homeostasis. Blood 129, 2124–2131. doi:10.1182/blood-2016-09-740563

Sipkins, D.A., Wei, X., Wu, J.W., Runnels, J.M., Côté, D., Means, T.K., Luster, A.D., Scadden, D.T., Lin, C.P., 2005. In vivo imaging of specialized bone marrow endothelial microdomains for tumour engraftment. Nature Publishing Group 435, 969–973. doi:10.1038/nature03703

Tamura, S., Suzuki-Inoue, K., Tsukiji, N., Shirai, T., Sasaki, T., Osada, M., Satoh, K., Ozaki, Y., 2016. Podoplanin-positive periarteriolar stromal cells promote megakaryocyte growth and proplatelet formation in mice by CLEC-2. Blood. doi:10.1182/blood-2015-08-663708

REVIEWERS' COMMENTS:

Reviewer #1 (Remarks to the Author):

The authors have been responsive to the comments.
They have added interesting data on aged bone marrow. They show no significant changes in the sinusoids, but could they comment on type H vessels or arterioles? Otherwise the paper is nice!

Reviewer #4 (Remarks to the Author):

The authors have addressed a number of the concerns raised, and even though the overall work remains descriptive and limited to only two bone marrow microenvironment components (CXCL12GFP cells and endothelial cells), I believe the manuscripts substantiate the important point that flow cytometry analyses capture only a very small proportion of bone marrow stroma cells and should therefore be complemented by microscopy-based analyses.

My major concern is that doubts remain, and are rightly raised by the authors themselves, on the nature of the GFP+ cells in the CXCL12 reporter mice used. If GFP reports CXCL12 expression, why are the authors sure that other cells produce CXCL12 too? Conversely, how can one be sure that all GFP+ cells do indeed produce high levels of CXCL12? I don't think this issue is a fundamental limitation, but I would expect the authors to discuss it somewhere in the manuscript, for example in the introduction where they motivate their studies and choice of reporters.

The analysis of bone marrow sinusoids in aged bone marrow is novel and very interesting, and deserves to be given full justice. For example, the Adams group has described in detail how endosteal vessels are affected by ageing, but these studies are not referenced in relation to the sinusoid work presented here. Together, these analyses suggest that more work will be needed to understand how space-limited changes in the microenvironment relate to dramatic changes in HSC function in aged animals. Again this point should be discussed, so that the presented work is fully put within context.

Minor point:
Please be consistent with nomenclature. Supp Fig 3 C labels Ly6a but the legend and most of the manuscript uses Sca-1.

Point-by point response to reviewers:

Reviewer #1 (Remarks to the Author):

The authors have been responsive to the comments.They have added interesting data on aged bone marrow. They show no significant changes in the sinusoids, but could they comment on type H vessels or arterioles? Otherwise the paper is nice!

Response: We appreciate the encouraging feedback and thank the reviewer for the positive comments. Regarding the question on type H vessels and arterioles, we did perform stainings that allowed us to visualize arterial and transitional vessel networks in the bone marrow of old mice. While visual inspection did suggest alterations of transitional vessels, the effects were subtle and hard to assess qualitatively. Unfortunately we do not have yet the tools for the automatic and highly specific classification and quantification of transitional vessels in 3D, so as to perform a nuanced and rigorous analysis, which would allow us to draw robust conclusions on the age-induced effects on these structures. We are currently working on the development of such computational tools, which we hope to use in the near future to address these and other pertinent questions.

Reviewer #4 (Remarks to the Author):

The authors have addressed a number of the concerns raised, and even though the overall work remains descriptive and limited to only two bone marrow microenvironment components (CXCL12GFP cells and endothelial cells), I believe the manuscripts substantiate the important point that flow cytometry analyses capture only a very small proportion of bone marrow stroma cells and should therefore be complemented by microscopy-based analyses.

Response: We are thankful to the reviewer for highlighting the relevance of our findings.

My major concern is that doubts remain, and are rightly raised by the authors themselves, on the nature of the GFP+ cells in the CXCL12 reporter mice used. If GFP reports CXCL12 expression, why are the authors sure that other cells produce CXCL12 too? Conversely, how can one be sure that all GFP+ cells do indeed produce high levels of CXCL12? I don't think this issue is a fundamental limitation, but I would expect the authors to discuss it somewhere in the manuscript, for example in the introduction where they motivate their studies and choice of reporters.

Response: We understand the reviewer's point and fully agree that currently it still remains unclear whether all bone marrow CXCL12-expressing cells are in fact faithfully labelled in CXCL12-GFP reporter mice. Our statement on the fact that most likely cells other than CARc express CXCL12 is based on a recent report from the Frenette group, mentioned previously, which suggests that GFP expression in these mice does not perfectly match cellular expression of CXCL12 at the protein level (Asada et al., 2017). These discrepancies are to some extent puzzling and point to a complex regulation of CXCL12 expression, presentation and secretion in different BM cell types.

However, what has been so far indeed well-established in the literature and is confirmed by our

own studies and observations is that: i) the so-called CXCL12-abundant reticular cell population, which is GFP^{bright} in CXCL12GFP reporter mice and LepR+, is a major source of CXCL12 in the bone marrow ii) deletion of CXCL12 expression in this subset causes major effects in hematopoiesis, including in the HSC progenitor population (Ding and Morrison, 2013; Greenbaum et al., 2013) iii) beyond CXCL12, CARc are the fundamental source of other critical factors, such as SCF and IL7 (Cordeiro Gomes et al., 2016; Ding et al., 2012) iv) both SECs and AECs express lower levels of CXCL12 than CARc, are thus labelled with very low/negligible GFP intensity by the CXCL12 reporter mice, which is barely detectable by confocal microscopy v) CXCL12GFP reporter mice are an optimal model to specifically visualize and study CARc.

Consequently, given that in our study we do not employ the reporter as a measurement of CXCL12 production but rather, exclusively as a marker of a relevant stromal cell population, the potential issues mentioned do not affect in any way our conclusions. In order to better clarify this point in our manuscript and following the reviewer's suggestion we have now edited the text in the Introduction and Results sections (changes highlighted in red):

Introduction: Although other mesenchymal stromal cells, which are not equally labelled in CXCL12 reporter mice, have been described to produce CXCL12 (Asada et al., 2017), CARc are essential to marrow function as they are additionally major sources of SCF and the pro-lymphoid cytokine interleukin-7 (IL-7)

Results: Hence, as previously reported, GFP^{bright} cells in *Cxcl12-Gfp* mice correspond exclusively to CD45^{-}Ter119^{-}CD31^{-}CD140b^{+} CARc (Omatsu et al., 2010), making this reporter strain the ideal model to specifically visualize and study this cell type via microscopy.

The analysis of bone marrow sinusoids in aged bone marrow is novel and very interesting, and deserves to be given full justice. For example, the Adams group has described in detail how endosteal vessels are affected by ageing, but these studies are not referenced in relation to the sinusoid work presented here. Together, these analyses suggest that more work will be needed to understand how space-limited changes in the microenvironment relate to dramatic changes in HSC function in aged animals. Again this point should be discussed, so that the presented work is fully put within context.

Response: We thank the reviewer for underlining the novelty of the new data on aged bone marrow, which we added in the last revision. We would like to clarify that the work from Kusumbe and colleagues (Adams group) was appropriately referenced and discussed in both the Introduction and Discussion of the previous version of the manuscript. The parts of the text where this was done are shown below:

Introduction: "Age-related disturbances in BM microenvironmental components are hypothesized to contribute to alterations in hematopoietic competence (Kovtonyuk et al., 2016) and include increased adipocyte infiltration (Ambrosi et al., 2017), perturbations of the cytokine milieu and reductions in bone volume and type H vessels (Kusumbe et al., 2016).

Discussion: "In contrast to the reported age-induced loss of type H vessels (Kusumbe et al., 2016), sinusoidal structures remain largely intact in the different BM regions and intrasinusoidal volumes were unchanged."

Following the reviewers suggestion we have modified one sentence of the discussion to reflect the importance of addressing in future studies how changes in BM microenvironment relate to HSC dysfunction during ageing.

"Future analyses should determine whether and to what extent the aged-induced perturbations of HSC function and hematopoiesis (Kovtonyuk et al., 2016) relate to spatial and structural changes in BM organization."

Minor point: Please be consistent with nomenclature. Supp Fig 3 C labels Ly6a but the legend and most of the manuscript uses Sca-1.

Response: We thank the reviewer for pointing this out. Indeed the accepted gene name is *Ly6a*, however, the most commonly used term in the field for the protein is Sca-1. We have clarified in the figure legend that Sca-1 and Ly6a are equivalent.

References

Ambrosi, T.H., Scialdone, A., Graja, A., Gohlke, S., Jank, A.-M., Bocian, C., Woelk, L., Fan, H., Logan, D.W., Schürmann, A., Saraiva, L.R., Schulz, T.J., 2017. Adipocyte Accumulation in the Bone Marrow during Obesity and Aging Impairs Stem Cell-Based Hematopoietic and Bone Regeneration. Stem Cell 1–21. doi:10.1016/j.stem.2017.02.009

Asada, N., Kunisaki, Y., Pierce, H., Wang, Z., Fernandez, N.F., Birbrair, A., Ma'ayan, A., Frenette, P.S., 2017. Differential cytokine contributions of perivascular haematopoietic stem cell niches. Nat. Cell Biol. 1–19. doi:10.1038/ncb3475

Cordeiro Gomes, A., Hara, T., Lim, V.Y., Herndler-Brandstetter, D., Nevius, E., Sugiyama, T., Tani-ichi, S., Schlenner, S., Richie, E., Rodewald, H.-R., Flavell, R.A., Nagasawa, T., Ikuta, K., Pereira, J.P., 2016. Hematopoietic Stem Cell Niches Produce Lineage-Instructive Signals to Control Multipotent Progenitor Differentiation. Immunity 45, 1219–1231. doi:10.1016/j.immuni.2016.11.004

Ding, L., Morrison, S.J., 2013. Haematopoietic stem cells and early lymphoid progenitors occupy distinct bone marrow niches. Nature 495, 231–235. doi:10.1038/nature11885

Ding, L., Saunders, T.L., Enikolopov, G., Morrison, S.J., 2012. Endothelial and perivascular cells maintain haematopoietic stem cells. Nature 481, 457–462. doi:10.1038/nature10783

Greenbaum, A., Hsu, Y.-M.S., Day, R.B., Schuettpelz, L.G., Christopher, M.J., Borgerding, J.N., Nagasawa, T., Link, D.C., 2013. CXCL12 in early mesenchymal progenitors is required for haematopoietic stem-cell maintenance. Nature 495, 227–230. doi:10.1038/nature11926

Kovtonyuk, L.V., Fritsch, K., Feng, X., Manz, M.G., Takizawa, H., 2016. Inflamm-Aging of Hematopoiesis, Hematopoietic Stem Cells, and the Bone Marrow Microenvironment. Front Immunol 7, 405–13. doi:10.3389/fimmu.2016.00502

Kusumbe, A.P., Ramasamy, S.K., Itkin, T., Mäe, M.A., Langen, U.H., Betsholtz, C., Lapidot, T., Adams, R.H., 2016. Age-dependent modulation of vascular niches for haematopoietic stem cells. Nature 532, 380–384. doi:10.1038/nature17638

Omatsu, Y., Sugiyama, T., Kohara, H., Kondoh, G., Fujii, N., Kohno, K., Nagasawa, T., 2010. The Essential Functions of Adipo-osteogenic Progenitors as the Hematopoietic Stem and Progenitor Cell Niche. Immunity 33, 387–399. doi:10.1016/j.immuni.2010.08.017
